# Metabolic Profiling and Gene Expression Analyses of Purple-Leaf Formation in Tea Cultivars (*Camellia sinensis* var. *sinensis* and var. *assamica*)

**DOI:** 10.3389/fpls.2021.606962

**Published:** 2021-03-05

**Authors:** Ming-zhi Zhu, Fang Zhou, Li-sha Ran, Yi-long Li, Bin Tan, Kun-bo Wang, Jian-an Huang, Zhong-hua Liu

**Affiliations:** ^1^Key Laboratory of Tea Science of Ministry of Education, College of Horticulture, Hunan Agricultural University, Changsha, China; ^2^National Research Center of Engineering Technology for Utilization of Functional Ingredients from Botanicals, Co-Innovation Center of Education Ministry for Utilization of Botanical Functional Ingredients, Hunan Agricultural University, Changsha, China; ^3^Research Center for Development and Utilization of Medicinal Plants in Eastern Hubei Province, Hubei University of Education, Wuhan, China; ^4^Tea Research Institute, Chinese Academy of Agricultural Sciences, Hangzhou, China

**Keywords:** purple-leaf tea cultivar, *Camellia sinensis*, anthocyanin, chlorophyll, biosynthetic pathway, metabolomics

## Abstract

Purple-leaf tea cultivars are known for their specific chemical composition that greatly influences tea bioactivity and plant resistance. Some studies have tried to reveal the purple-leaf formation mechanism of tea by comparing the purple new leaves and green older leaves in the same purple-leaf tea cultivar. It has been reported that almost all structural genes involved in anthocyanin/flavonoid biosynthesis were down-regulated in purple-leaf tea cultivars when the purple new leaves become green older leaves. However, anthocyanin/flavonoid biosynthesis is also affected by the growth period of tea leaves, gradually decreasing as new tea leaves become old tea leaves. This leads to uncertainty as to whether the purple-leaf formation is attributed to the high expression of structural genes in anthocyanin/flavonoid biosynthesis. To better understand the mechanisms underlying purple-leaf formation, we analyzed the biosynthesis of three pigments (chlorophylls, carotenoids, and anthocyanins/flavonoids) by integrated metabolic and gene expression analyses in four purple-leaf tea cultivars including *Camellia sinensis* var. *sinensis* and var. *assamica*. Green-leaf and yellow-leaf cultivars were employed for comparison. The purple-leaf phenotype was mainly attributed to high anthocyanins and low chlorophylls. The purple-leaf phenotype led to other flavonoid changes including lowered monomeric catechin derivatives and elevated polymerized catechin derivatives. Gene expression analysis revealed that 4-coumarate: CoA ligase (*4CL*), anthocyanidin synthase (*ANS*), and UDP-glucose: flavonoid 3-O-glucosyltransferase (*UFGT*) genes in the anthocyanin biosynthetic pathway and the uroporphyrinogen decarboxylase (*HEME*) gene in the chlorophyll biosynthetic pathway were responsible for high anthocyanin and low chlorophyll, respectively. These findings provide insights into the mechanism of purple-leaf formation in tea cultivars.

## Introduction

The tea plant [*Camellia sinensis* (L.) O. Kuntze], cultivated for the production of non-alcoholic beverages, is an economically important woody crop worldwide (Zhu et al., 2017, 2019, 2020b; Yang et al., 2019). Leaf color is an important agronomic trait of this plant. Tea leaves are predominantly green, but various tea cultivars exhibiting leaf color variations (e.g., purple-leaf, yellow-leaf, and albino-leaf cultivars) have been developed through long-term natural hybridization and artificial selection (Wang et al., 2016; Li et al., 2018a; Shen et al., 2018). Among these tea cultivars, purple-leaf tea is attracting increasing attention due to its delightful, unique color, and multiple health benefits (Zhou et al., 2017). As a result, several new tea cultivars with purple leaves have recently been developed in China, Japan, India, and Kenya (Terahara et al., 2001; Saito et al., 2011; Kerio et al., 2012; Jiang et al., 2013; Joshi et al., 2015; Lai et al., 2016).

Numerous studies have shown that the leaf color of a tea plant is attributed to three major classes of pigment: chlorophylls, carotenoids, and flavonoids (Shen et al., 2018). Chlorophylls correspond to the green pigments in leaves, whereas carotenoids are generally responsible for orange, yellow, and red colors (Zhao and Tao, 2015). Flavonoids are a large class of secondary metabolites, which comprise anthocyanins, flavanols, flavonols, flavones, and proanthocyanidins (Winkel-Shirley, 2001). Anthocyanins, the most conspicuous class of flavonoids in the plant kingdom, are important plant pigments responsible for red, pink, purple, and blue colors (Zhao and Tao, 2015). The purple-leaf phenotype in tea plants is typically associated with high anthocyanin accumulation (Sun et al., 2016); the anthocyanin content in purple-leaf tea is approximately three times higher than that in green-leaf tea (Wei et al., 2016). Moreover, as a key factor responsible for the striking purple color in tea leaves, anthocyanins play a critical role in protecting plants against various biotic and abiotic stresses, such as pathogen infection, wounding, ultraviolet-B radiation, drought, cold, and nutrient deficiency (Kim et al., 2017). Furthermore, anthocyanins have multiple health-promoting effects, such as antioxidative, cholesterol-lowering, antihypertensive, anti-aging, neuroprotective, and anticarcinogenic properties (Blesso, 2019). Thus, anthocyanin accumulation improves the health-related biological functions of tea, thereby enhancing tea quality and increasing its demand by consumers. As such, anthocyanin-rich purple-leaf tea cultivars have become a highly significant breeding target (Kumari et al., 2020).

**FIGURE 1:**
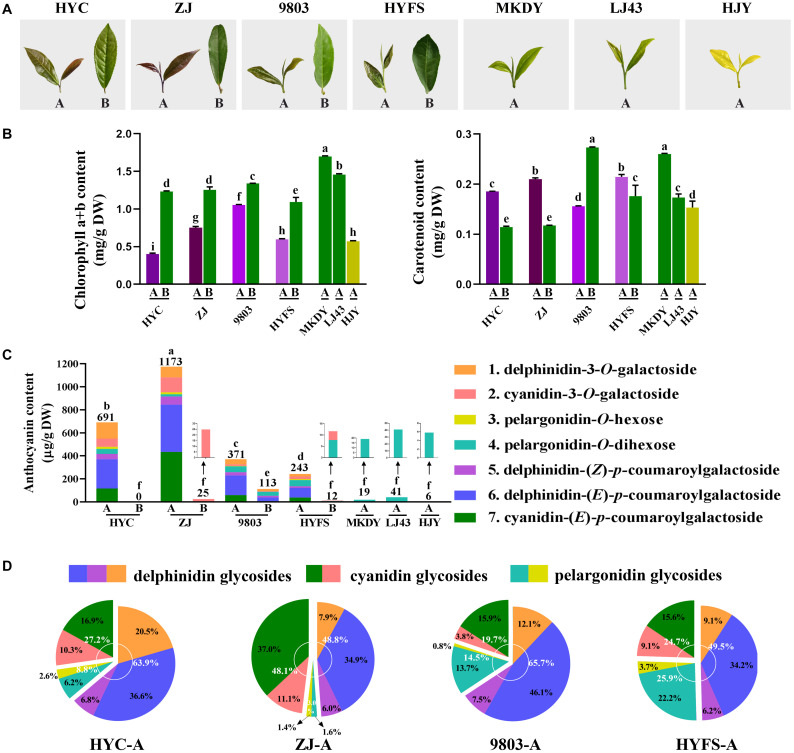
**(A)** Growth performance and **(B)** chlorophyll a, chlorophyll b, and carotenoid content changes in the leaves of purple-leaf, green-leaf, and yellow-leaf tea cultivars. Values in the same row that are labeled with different letters (a–i) differ significantly (*p* < 0.05). **(C)** Anthocyanin content. Values in the same row that are labeled with different letters (a–f) differ significantly (*p* < 0.05). **(D)** Proportion of anthocyanins in the new leaves of purple-leaf tea cultivars. The letters A and B after the tea cultivar name represent new leaves (one bud and two leaves) and old leaves, respectively.

Anthocyanin accumulation, which originates from a sub-pathway of flavonoid biosynthesis in the phenylpropanoid metabolism, has been thoroughly characterized in model plants such as *Arabidopsis thaliana*, *Zea mays*, and *Petunia hybrida* (Appelhagen et al., 2014). Anthocyanin biosynthesis is controlled by a series of structural genes. Genomic and transcriptomic analyses have indicated that the anthocyanin biosynthetic pathway is also conserved in the tea leaf (Li et al., 2017; Wei et al., 2018; Shi et al., 2020). The leaf color of anthocyanin-rich tea cultivars is not always purple, with purple-colored new leaves gradually turning green as they age (Figure 1A). Research on the color transition of purple-leaf tea cultivars during leaf development has made significant progress (Li et al., 2017; Wang et al., 2017; Shen et al., 2018). For example, Shen et al. (2018) reported that almost all structural genes involved in anthocyanin/flavonoid biosynthesis were down-regulated in purple-leaf tea cultivars when the purple new leaves become green older leaves, indicating that almost all structural genes are related to purple-leaf formation in tea leaves. Other studies obtained similar conclusions (Li et al., 2017; Wang et al., 2017). However, anthocyanin/flavonoid biosynthesis is affected by the growth period of tea leaves, gradually decreasing as new tea leaves become old tea leaves (Wan, 2008). This leads to uncertainty as to whether the color transition is attributed to the decreased expression of structural genes in anthocyanin/flavonoid biosynthesis. Further, the genetic background of various tea cultivars is complicated, resulting in increased complexity of pigment biosynthesis (Wei et al., 2018). Thus, the molecular mechanism underlying purple-leaf formation in tea cultivars remains unknown.

Tea leaves are also rich in other types of flavonoids besides anthocyanins, such as flavanols, flavonols, flavones, and proanthocyanidins. Flavonoids account for 18-30% of the dry weight (DW) in green tea, among which various flavan-3-ols, known as catechin derivatives, represent 60-80% of the total flavonoids in green tea (Zhu et al., 2020a). These catechin derivatives mainly consist of epigallocatechin-3-*O*-gallate (EGCG), epicatechin-3-*O*-gallate (ECG), epigallocatechin (EGC), epicatechin (EC), gallocatechin-3-*O*-gallate (GCG), gallocatechin (GC), and catechin (Zhu et al., 2017). Catechin derivatives, especially EGCG, are the major taste and bioactive compounds in tea, which have various health benefits, including antioxidant, anti-cancer, anti-bacterial, anti-viral, and anti-obesity effects (Zhu et al., 2019). In addition, numerous flavonols, flavones, and proanthocyanidins also contribute to the taste and health benefits of tea (Zhu et al., 2017, 2020a). Furthermore, these flavonoids share the same synthetic pathway with anthocyanin. We therefore hypothesize that the biosynthesis of these flavonoids is affected by the anthocyanin-rich nature of purple-leaf tea cultivars, leading to differences in biological activity between purple-leaf and non-purple-leaf tea cultivars (Shen et al., 2018). However, the biosynthesis of these flavonoids in anthocyanin-rich purple-leaf tea cultivars remain unclear.

The purpose of this study is to investigate the three main pigments (chlorophylls, carotenoids, and anthocyanins/flavonoids) in four purple-leaf tea cultivars, and explore the molecular mechanism underlying purple-leaf formation in tea cultivars through integrated metabolic and gene expression analyses. The four purple-leaf tea cultivars vary from light to dark purple. The old green leaves of the four purple-leaf tea cultivars, two green-leaf tea cultivars, and one yellow-leaf tea cultivar are employed as a comparison. The results of this study provide new insights into purple-leaf formation in tea cultivars.

## Materials and Methods

### Chemicals and Plant Materials

Acetonitrile, methanol, water, and formic acid (FA) were purchased from Merck (Darmstadt, Germany). Ethanol was provided by Sinopharm Chemical Reagent Co., Ltd. (Shanghai, China). The standards of EGCG, ECG, EGC, EC, GCG, GC, catechin, gallic acid, caffeine, theobromine, theophylline, theanine, apigenin-6-*C*-glucoside-8-*C*-arabinoside (isoschaftoside), myricitin-3-*O*-galactoside, myricitin-3-*O*-glucoside, apigenin-8-*C*-glucoside (vitexin), apigenin-6-*C*-glucoside (isovitexin), quercetin-3-*O*-galactoside, quercetin-3-*O*-glucoside, rutin, kaempferol-3-*O*-galactoside, kaempferol-3-*O*-glucoside, kaempferol, quinic acid, ellagic acid, delphinidin, cyanidin, pelargonidin, delphinidin-3-*O*-galactoside, and cyanidin-3-*O*-galactoside were obtained from Shanghai Tauto Biotech Co., Ltd. (Shanghai, China), and their purities were >95%.

Seven tea cultivars including Hongyecha (HYC), Zijuan (ZJ), 9803, Hongyafoshou (HYFS), Longjing 43 (LJ43), Huangjinya (HJY), and Mengkudaye (MKDY) were used in this study (Figure 1A). HYC, ZJ, 9803, and HYFS are purple-leaf cultivars; the first two have dark purple new leaves and the second two have light purple new leaves. These new leaves gradually turn green as they grow older. LJ43 and MKDY are green-leaf cultivars, whereas HJY is a yellow-leaf cultivar. ZJ and MKDY are *Camellia sinensis* var. *assamica* (CSA) cultivars originating from Yunnan Province, southwest China, whereas HYC, 9803, HYFS, LJ43, and HJY are *Camellia sinensis* var. *sinensis* (CSS) cultivars. HYC, ZJ, LJ43, and HJY were collected from the Tea Germplasm Repository of Tea Research Institute, Hunan Academy of Agricultural Sciences (28°48′ N, 113°36′ E, Changsha, Hunan, China); 9803, HYFS, and MKDY were collected from the Tea Germplasm Repository of Hunan Agricultural University (28°16′ N, 113°24′ E, Changsha, Hunan, China). The new leaves (one bud and two leaves, A) and old leaves (B) were harvested on April 5, 2018, and three independent biological replicates were performed. Each replicate was collected from 15 randomly selected tea plants. The samples were frozen in liquid nitrogen then stored at −80°C for further analysis.

### Measurement of Chlorophylls and Carotenoids in the Tea Leaves

0.1 g of freeze-dried and crushed sample was extracted with 15 mL of 95% ethanol and then incubated for 48 h in the dark. The extract was filtered and measured spectrophotometrically at 665 nm for chlorophyll a, 649 nm for chlorophyll b, and 470 nm for carotenoids. The chlorophyll a, chlorophyll b, and carotenoid content was determined following a previously described method (Song et al., 2017). Each sample were analyzed in triplicate.

### Ultra-Performance Liquid Chromatography Coupled With Diode-Array Detector and Quadrupole/Time of Flight Tandem Mass Spectrometry (UPLC-DAD-QTOF-MS) Analysis

The UPLC-DAD-QTOF-MS analysis was carried out according to our previously described method, with slight modifications (Zhu et al., 2017). 0.1 g of freeze-dried and crushed sample was ultrasonically extracted for 40 min with 5 mL of 70% methanol containing 0.1% FA. The extract was centrifuged at 12,000 *g* for 10 min, and then the supernatant was diluted with 70% methanol containing 0.1% FA. The solution was filtered through a 0.22 μm membrane (ANPEL Laboratory Technologies Inc., Shanghai, China), and then analyzed using an UPLC-DAD-QTOF-MS system consisting of a 1290 Infinity UPLC System, a DAD detector, and a 6530 Infinity Jet Stream ESI-Q-TOF system (Agilent Technologies, Inc., Santa Clara, CA, United States). An ACQUITY UPLC HSS T3 column (1.8 μm, 2.1 mm × 150 mm; Waters, Milford, MA, United States) was used for the reversed phase separation. The column temperature was maintained at 45°C. The mobile phase contained solvent A (water with 0.1% FA) and solvent B (acetonitrile), and the flow rate of the mobile phase was 0.3 mL/min. Gradient elution procedures were set as follows: 0–5 min, 98 to 95% A; 5–20 min, 95 to 80% A; 20–25 min, 80 to 65% A; 25–28 min, 65 to 0% A; 28–30 min, 0% A. Five minutes of post-run re-equilibration was conducted before the next injection. The DAD detector was set at 278, 350, and 520 nm for acquiring chromatograms, and UV/Vis spectra were recorded in the range of 200–600 nm. The QTOF MS system was operated in positive ion (PI) mode and negative ion (NI) mode. The MS parameters were set as follows: dry gas temperature, 345°C; drying gas flow, 10 L/min; nebulizer, 50 psig; sheath gas temperature, 350°C; sheath gas flow, 11 L/min; the capillary voltage was +4 kV and −4 kV for PI mode and NI mode, respectively. The QTOF MS system was operated across the range of 100–1200 *m/z* in full scan mode for relative qualitative analyses. For MS/MS detection, all precursors were fragmented using 20–40 eV voltage. Each sample were analyzed in triplicate. In addition, a quality control (QC) sample was established by blending an equal volume of extract from each biological sample, and the QC sample were measured after every 10 samples to evaluate the stability of the UPLC-DAD-QTOF-MS system.

### Identification and Measurement of Anthocyanins

The acid hydrolysis was adopted to acquire the anthocyanin aglycones in the tea leaves according to the method described by Lai et al. (2016). Then the anthocyanin aglycones were identified with the corresponding standards by using UPLC-DAD-QTOF-MS. Subsequently, the anthocyanins in the tea leaves were identified by UPLC-DAD-QTOF-MS, and the partial results were further confirmed by the corresponding standards. The quantification of anthocyanins was determined by a UPLC-DAD calibration curve of standard cyanidin-3-*O*-glucoside at 520 nm. The results were expressed as mg of cyanidin-3-*O*-galactoside equivalents per g DW of tea leaves.

### Determination of Major Chemical Constituents in Tea Leaves

The EGCG, ECG, EGC, EC, GCG, catechin, gallic acid, theanine, caffeine, theobromine, and theophylline content was measured by high pressure liquid chromatography with ultraviolet detection (HPLC-UV), as described previously (Gong et al., 2020). All analysis was performed in triplicate.

### Quantitative Reverse Transcriptase-Polymerase Chain Reaction (qRT-PCR) Analysis

Total RNAs were extracted from the tea leaves using the RN09-EASYspin Plant RNA Extraction Kit (Aidlab Biotechnologies Co., Ltd., Beijing, China), and reverse transcribed with PrimeScript^TM^ RT Reagent Kit (Takara Bio. Inc., Dalian, China). qPCR was performed in triplicate on the QuantStudio 3 Real-Time PCR System (Applied Biosystems, Carlsbad, CA, United States) by using the TB Green^TM^ Premix Ex Taq^TM^ II Kit (Takara Bio. Inc., Dalian, China). The primers of the targeted genes involved in the chlorophyll, carotenoid, anthocyanin/flavonoid synthesis were purchased from TSINGKE Biology Co., Ltd. (Beijing, China), and their sequences were shown in Supplementary Table 1. The genes in chlorophyll biosynthesis included glutamyl-tRNA reductase (*HEMA*), glutamate 1-semialdehyde aminotransferase (*HEML*), uroporphyrinogen decarboxylase (*HEME*), coproporphyrinogen oxidative decarboxylase (*HEMF*), ferrochelatase (*FECH*), Mg chelatase H subunit (*CHLH*), protochlorophyllide oxidoreductase (*POR*), chlorophyllide a oxygenase (*CAO*), and NYC1-like (*NOL*) genes. The genes in carotenoid synthesis included phytoene synthase (*PSY*), phytoene desaturase (*PDS*), ζ-carotene desaturase (*ZDS*), and zeaxanthin epoxidase (*ZEP*) genes. The genes in anthocyanin/flavonoid synthesis included phenylalanine ammonia lyase (*PAL*), cinnamate-4-hydroxylase (*C4H*), 4-coumarate: CoA ligase (*4CL*), chalcone synthase (*CHS*), chalcone isomerase (*CHI*), flavanone-3-hydroxylase (*F3H*), flavonoid 3′-hydroxylase (F3′H), flavone synthase (*FNS*), flavonol synthase (*FLS*), dihydroflavonol 4-reductase (DFR), anthocyanidin synthase (ANS), anthocyanidin reductase (*ANR*), leucoanthocyanidin reductase (*LAR*), and UDP-glucose: flavonoid glucosyltransferase (*UFGT*). Reference gene of glyceraldehyde-3-phosphate dehydrogenase (*GAPDH*) were used as an internal control to quantify the relative transcript levels of the target genes in each sample (Shen et al., 2018).

### Data Analysis

Statistical analysis was performed with GraphPad Prism 7.04 (GraphPad Software Inc., La Jolla, CA, United States), and data are presented as mean ± standard deviation (SD). Data sets involving more than two groups were assessed by one-way analysis of variance (ANOVA) followed by Fisher’s least significant difference (LSD) tests.

## Results

### The Chlorophylls and Their Synthetic Genes in Purple-Leaf Tea Cultivars

The chlorophyll content in the tea leaves is shown in Figure 1B. The level of chlorophyll a + b in the new leaves of purple-leaf tea cultivars (NL-PTC) was dramatically lower than that in the old leaves of purple-leaf tea cultivars (OL-PTC). Furthermore, the level of chlorophyll a + b in NL-PTC was also lower than that in the new leaves of green-leaf tea cultivars (NL-GTC). In addition, the level of chlorophyll a + b in the new leaves of the yellow-leaf tea cultivar (NL-YTC) (which is a well-known chlorophyll-deficient chlorina tea cultivar) was lower than that in the green tea leaves, including NL-GTC and OL-PTC; this is consistent with previous findings (Wang et al., 2014).

**FIGURE 2:**
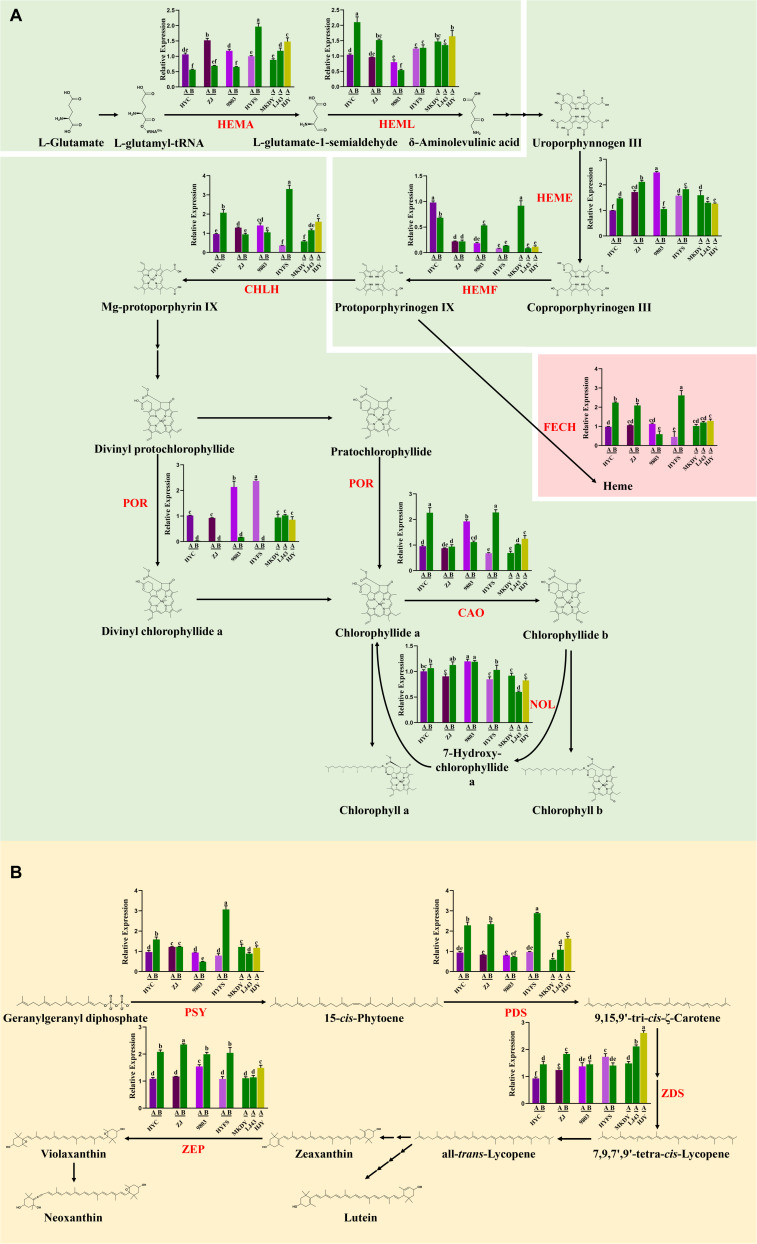
Changes of gene expression involving chlorophyll biosynthesis **(A)** and carotenoid biosynthesis **(B)** in the leaves of purple-leaf, green-leaf, and yellow-leaf tea cultivars. Data were assessed by one-way ANOVA followed by Fisher’s LSD test. Values in the same row that are labeled with different letters (a–f) differ significantly (*p* < 0.05). The letters A and B after the tea cultivar name represent new leaves (one bud and two leaves) and old leaves, respectively. HEMA, glutamyl-tRNA reductase; HEML, glutamate 1-semialdehyde aminotransferase; HEME, uroporphyrinogen decarboxylase; HEMF, coproporphyrinogen oxidative decarboxylase; FECH, ferrochelatase; CHLH, Mg chelatase H subunit; POR, protochlorophyllide oxidoreductase; CAO, chlorophyllide a oxygenase; NOL, NYC1-like; PSY, phytoene synthase; PDS, phytoene desaturase; ZDS, ζ-carotene desaturase; ZEP, zeaxanthin epoxidase.

We compared the expression patterns of nine key genes in chlorophyll biosynthesis (Figure 2A). *HEMA* and *HEML* are key genes in phase I of chlorophyll biosynthesis (Beale, 2005). The *HEMA* expression of new leaves was higher than that of old leaves in the same purple-leaf tea cultivar, except for the HYFS cultivar, but no consistent observation of the relative level of *HEMA* expression was made between NL-PTC and NL-GTC. Expression of the *HEML* gene in NL-PTC was lower than that in NL-GTC except that no difference was observed between HYFS-A and NL-GTC. *HEME* and *HEMF* genes are involved in phase II of chlorophyll biosynthesis (Beale, 2005). The *HEME* expression of new leaves was slightly lower than that of old leaves in the same purple-leaf tea cultivar, except for the 9803 cultivar. Nevertheless, no consistent observation of the relative level of *HEMF* expression was made between NL-PTC and NL-GTC, or between new leaves and old leaves in the same purple-leaf tea cultivar.

*CHLH*, *FECH*, *POR*, *CAO*, and *NOL* genes are related to phase III of chlorophyll biosynthesis (Beale, 2005). The *NOL* expression of new leaves was lower than or equal to that of old leaves in the same purple-leaf tea cultivar. However, expression of the *NOL* gene in NL-PTC was higher than or equal to that in NL-GTC. Expression of the *POR* gene in NL-PTC was remarkably higher than that in OL-PTC, and higher than or equal to that in NL-GTC. Further, the *FECH* expression of new leaves was lower than that of old leaves in the same purple-leaf tea cultivar, except for the 9803 cultivar. In addition, no consistent observations of the relative levels of *CHLH* and *CAO* expression were made between NL-PTC and NL-GTC, or between new leaves and old leaves in the same purple-leaf tea cultivar.

### The Carotenoids and Their Synthetic Genes in Purple-Leaf Tea Cultivars

The level of carotenoids in the tea leaves is shown in Figure 1B. A significant difference was observed between LJ43-A and MKDY-A. The level of carotenoids in NL-PTC was lower than that in MKDY-A, but higher than or equal to that in LJ43-A, except for 9803-A. In addition, the carotenoid content of new leaves was higher than that of old leaves in the same purple-leaf tea cultivar, except for the 9803 cultivar.

The expression patterns of four key genes, including *PSY*, *PDS*, *ZDS*, and *ZEP* in the carotenoid biosynthetic pathway, were measured to reveal the carotenoid biosynthesis in the purple-leaf tea cultivars (Figure 2B; Song et al., 2017). A significant difference in the expression of these carotenoid biosynthetic genes was observed between LJ43-A and MKDY-A, except for the *ZEP* gene. Further, only expression of the *PSY* gene in MKDY-A was higher than that in LJ43-A. Moreover, expression of the *PSY* gene in NL-PTC was lower than that in MKDY-A, but no difference was observed between ZJ-A and MKDY-A. Meanwhile, expression of the *PSY* gene in NL-PTC was higher than or equal to that in LJ43-A.

### Metabolic Profiling of Anthocyanin/Flavonoid in Purple-Leaf Tea Cultivars

We used a previously established non-targeted metabolomics approach to profile anthocyanin/flavonoid in the tea leaves with the UPLC-DAD-QTOF-MS system (Zhu et al., 2017). A total of 87 flavonoids were identified, as well as 12 phenolic acids, five amino acids, two alkaloids, one organic acid, six nucleosides, and three carbohydrates, based on a comparison of retention times, MS and MS/MS spectra with standards, metabolome databases, and/or references (Table 1; Zhu et al., 2017). These flavonoids included six anthocyanins, 20 monomeric catechin derivatives, 36 polymerized catechin derivatives, and 25 flavonols, flavones, and their glycosides. The levels of six anthocyanins and six major monomeric catechin derivatives were also measured by UPLC-DAD and HPLC-UV, respectively.

**TABLE 1:** Relative fold change of compounds in the leaves of purple-leaf, green-leaf, and yellow-leaf tea cultivars according to UPLC-QTOF-MS analysis*.

ID	*t*_*R*_ (min)	Formula	Name	Fold change (tea leaves versus LJ43-A)^#^										
				HYC-A	HYC-B	ZJ-A	ZJ-B	9803-A	9803-B	HYFS-A	HYFS-B	MKDY-A	LJ43-A	HJY- A
**Monomeric catechin derivatives**														
1	7.275	C_21_H_24_O_12_	Gallocatechin-glucoside isomer 1	1.55^a^	0.35^f^	0.00^g^	0.00^g^	1.59^a^	1.32^b^	1.20^c^	0.91^e^	1.24^c^	1.00^d^	0.00^g^
2	7.649	C_15_H_14_O_7_	Gallocatechin (GC)	3.90^a^	0.68^f^	1.44^d^	0.31^g^	0.66^f^	0.41^g^	0.00^h^	3.47^c^	3.79^b^	1.00^e^	0.00^h^
3	8.586	C_21_H_24_O_12_	Gallocatechin-glucoside isomer 2	0.91^e^	0.51^h^	1.24^c^	0.30^i^	0.85^f^	0.90^ef^	2.04^a^	2.02^a^	0.86^f^	1.00^d^	0.80^g^
4	8.852	C_15_H_14_O_7_	Epigallocatechin (EGC)^§,†^	1.21^c^	0.28^g^	1.73^a^	0.08^h^	0.33^g^	0.52^f^	1.46^b^	0.63^e^	1.78^a^	1.00^d^	1.50^b^
5	9.284	C_15_H_14_O_6_	Catechin^§,†^	4.60^a^	0.48^gh^	4.62^a^	0.36^h^	0.64^g^	1.76^d^	3.28^b^	0.36^h^	2.39^c^	1.00^f^	1.28^e^
6	9.538	C_21_H_24_O_12_	Gallocatechin-glucoside isomer 3	1.13^ef^	0.29^g^	1.35^d^	0.25^g^	1.03^f^	1.49^c^	1.68^b^	1.87^a^	1.22^de^	1.00^f^	1.84^a^
7	10.637	C_15_H_14_O_6_	Epicatechin (EC)^§,†^	0.95^d^	0.19^g^	1.65^b^	0.15^gh^	0.60^f^	0.11^h^	1.04^c^	0.55f	0.84^e^	1.00^c^	1.90^a^
8	10.784	C_22_H_18_O_11_	Epigallocatechin-3-*O*-gallate (EGCG)^§,†^	0.67^d^	0.17^g^	1.00^b^	0.03^h^	0.47^e^	0.36^f^	0.84^c^	0.20^g^	1.71^a^	1.00^b^	0.86^c^
9	10.869	C_24_H_20_O_10_	Epigallocatechin-caffeoate isomer 1	1.60^d^	0.44^g^	1.38^e^	3.53^b^	1.64^d^	1.98^c^	0.00^i^	0.00^i^	0.29^h^	1.00^f^	9.59^a^
10	11.382	C_24_H_20_O_10_	Epigallocatechin-caffeoate isomer 2	0.35^b^	0.62^b^	0.37^b^	0.44^b^	0.85^b^	0.67^b^	1.61^b^	1.01^b^	0.35^b^	1.00^b^	5.72^a^
11	12.585	C_15_H_14_O_5_	Epiafzelechin	0.70^d^	0.13^g^	0.59^e^	0.02^h^	0.35^f^	0.33^f^	0.66^de^	0.14^g^	1.48^b^	1.00^c^	1.84^a^
12	12.695	C_23_H_20_O_11_	Epigallocatechin-3-*O*-(4″-*O*-methyl)-gallate (4″-Me-EGCG)	0.86^b^	0.32^e^	0.00^f^	0.00^f^	0.67^c^	0.49^d^	0.95^a^	0.82^b^	0.00^f^	1.00^a^	0.62^c^
13	13.368	C_23_H_20_O_11_	Epigallocatechin-3-*O*-(3″-*O*-methyl)-gallate (3″-Me-EGCG)	0.31^f^	0.29^fg^	26.94^a^	1.95^d^	0.36^f^	0.25^g^	0.27^fg^	0.28^fg^	13.95^b^	1.00^e^	7.47^c^
14	13.924	C_23_H_20_O_11_	Gallocatechin-3-*O*-(4″-*O*-methyl)-gallate (4″-Me-GCG)	0.00^e^	0.06^e^	0.00^e^	0.00^e^	0.49^d^	0.67^c^	0.62^c^	0.00^e^	0.00^e^	1.00^b^	1.17^a^
15	14.587	C_22_H_18_O_10_	Epicatechin-3-*O*-gallate (ECG)^§,†^	0.63^f^	0.21^gh^	1.49^a^	0.02^j^	0.17^h^	0.12^i^	0.91^e^	0.24^g^	0.95^d^	1.00^c^	1.06^b^
16	15.148	C_22_H_18_O_9_	Epigallocatechin-3-*O*-(4-hydroxybenzoate)	1.03^g^	1.33^f^	1.73^d^	0.71^i^	1.05^g^	0.80^e^	1.65^e^	2.35^b^	2.94^a^	1.00^g^	2.12^c^
17	15.410	C_23_H_20_O_11_	Gallocatechin-3-*O*-(3″-*O*-methyl)-gallate (3″-Me-GCG)	1.18^a^	0.26^f^	0.52^d^	0.03^h^	0.65^c^	0.46^e^	0.25^f^	0.14^g^	0.21^f^	1.00^b^	0.51^de^
18	16.330	C_24_H_20_O_10_	Epigallocatechin-caffeoate isomer 3	2.46^f^	2.49^f^	12.89^a^	0.82^h^	0.79^h^	0.00^i^	4.51^b^	3.97^d^	2.97^e^	1.00^g^	4.09^c^
19	18.352	C_22_H_18_O_9_	Epiafzelechin-3-*O*-gallate	0.75^e^	0.10^h^	0.38^f^	0.01^i^	0.22^g^	0.18^g^	0.87^d^	0.08^h^	2.49^a^	1.00^c^	1.21^b^
20	18.406	C_23_H_20_O_10_	Epicatechin-3-*O*-(3″-*O*-methyl)-gallate (3″-Me-ECG)	0.02^h^	0.06^g^	42.42^a^	4.98^d^	0.09^g^	0.00^h^	0.19^f^	0.00^h^	10.29^c^	1.00^e^	11.52^b^
**Polymerized catechin derivatives**														
21	5.822	C_30_H_26_O_15_	Dehydrotheasinensin C isomer 1	0.37^g^	0.21^h^	0.49^f^	0.08^i^	0.94^d^	1.35^a^	0.57^e^	1.22^b^	0.89^d^	1.00^c^	0.69^d^
22	8.030	C_30_H_26_O_15_	Dehydrotheasinensin C isomer 2	0.69^g^	0.20^i^	1.06^d^	0.26^h^	1.04^d^	1.41^b^	0.67^g^	1.73^a^	1.16^c^	1.00^e^	0.94^f^
23	8.180	C_30_H_26_O_14_	Theasinensin C	1.39^f^	2.20^d^	0.83^i^	1.43^e^	0.64^j^	0.43^k^	1.05^g^	22.01^b^	4.52^c^	1.00^h^	25.81^a^
24	8.430	C_37_H_30_O_18_	Theasinensin B	0.74^c^	0.09^g^	0.63^d^	0.02^h^	1.06^ab^	1.07^a^	0.96^b^	0.24^f^	0.71^c^	1.00^b^	0.55^e^
25	8.470	C_30_H_26_O_13_	Theasinensin E	3.28^f^	4.26^e^	19.90^a^	7.06^b^	2.41 ^g^	1.54^i^	2.37^g^	6.75^c^	2.21^h^	1.00^j^	4.63^d^
26	8.603	C_45_H_38_O_18_	Procyanidin C isomer 1	1.12^d^	0.00^h^	1.09^d^	0.00^h^	2.48^a^	0.68^f^	1.32^b^	1.23^bc^	0.54^g^	1.00^e^	1.16^c^
27	8.711	C_30_H_26_O_15_	Dehydrotheasinensin C isomer 3	1.28^d^	0.18^k^	0.95^i^	0.31^j^	1.46^c^	1.23^e^	1.08^g^	2.02^a^	1.13^f^	1.00^h^	1.64^b^
28	8.736	C_30_H_26_O_13_	Gallocatechin-(4α→ 8)-epicatechin	1.99^g^	5.19^b^	2.73^f^	4.63^c^	0.65^j^	0.32^k^	0.80^i^	22.33^a^	3.54^d^	1.00^h^	3.39^e^
29	8.761	C_30_H_26_O_12_	Procyanidin B2	2.02^c^	0.40^h^	2.95^a^	0.70^g^	0.78^f^	0.30^i^	1.30^d^	0.00^j^	1.26^d^	1.00^e^	2.19^b^
30	9.035	C_30_H_26_O_12_	Procyanidin B3	1.86^b^	0.28^fg^	1.76^b^	0.30^f^	0.71^e^	0.21^fg^	1.26^c^	0.16^g^	1.04^d^	1.00^d^	2.67^a^
31	9.234	C_45_H_38_O_18_	Procyanidin C isomer 2	2.65^b^	0.68^j^	2.90^a^	1.15^g^	1.04^h^	0.61^k^	1.50^e^	1.67^a^	1.21^f^	1.00^i^	2.53^c^
32	9.724	C_44_H_32_O_22_	Theacitrin C	1.57^d^	0.19^j^	0.69^g^	0.02^k^	1.78^c^	1.89^b^	2.06^a^	0.36^h^	0.83^f^	1.00^e^	0.30^i^
33	9.732	C_44_H_34_O_22_	Theasinensin A	1.38^d^	0.11^h^	0.58^f^	0.02^i^	1.61^c^	1.37^d^	1.87^b^	0.26^g^	8.55^a^	1.00^e^	0.29^g^
34	9.757	C_30_H_26_O_12_	Procyanidin B5	1.70^d^	0.33^j^	1.09^f^	0.45^i^	0.74^h^	0.33^j^	1.45^e^	2.12^b^	1.98^c^	1.00^g^	2.63^a^
35	9.989	C_37_H_30_O_17_	Epigallocatechin-(4β→ 8)-epicatechin-3-*O*-gallate	3.13^d^	4.58^c^	7.16^b^	2.41^de^	0.90^gh^	0.52^h^	1.84^ef^	11.89^a^	1.54^fg^	1.00^gh^	1.42^fg^
36	10.222	C_45_H_38_O_18_	Procyanidin C isomer 3	1.71^b^	0.20^i^	1.39^c^	0.43^g^	0.89^f^	0.30^h^	1.07^e^	1.03^e^	1.21^d^	1.00^e^	2.56^a^
37	10.330	C_37_H_30_O_17_	Epicatechin-(4β→ 8)-epigallocatechin-3-*O*-gallate	3.36^b^	1.58^de^	15.00^a^	1.50^de^	1.23^ef^	0.66^g^	1.87^cd^	2.04^c^	0.73^g^	1.00^fg^	2.21^c^
38	10.720	C_44_H_34_O_22_	Theasinensin D	1.55^a^	0.00^g^	0.98^c^	0.01^g^	1.22^b^	0.95^cd^	1.50^a^	0.41^f^	0.90^d^	1.00^c^	0.52^e^
39	10.811	C_44_H_36_O_22_	Assamicain A	1.33^c^	0.17^i^	0.85^f^	0.08^j^	1.42^b^	1.19^d^	1.72^a^	0.40^h^	1.31^c^	1.00^e^	0.72^g^
40	10.911	C_44_H_34_O_21_	GCG-(4β→ 8)-ECG	1.60^b^	0.37^g^	1.60^b^	0.15^i^	1.37^a^	1.45^c^	20.68^a^	0.58^f^	0.36^g^	1.00^e^	0.29^h^
41	10.969	C_44_H_32_O_22_	Samarangenin B	1.25^d^	0.00^i^	0.62^f^	0.02^i^	1.36^c^	1.44^b^	1.52^a^	0.30^h^	0.43^g^	1.00^e^	0.28^h^
42	11.077	C_37_H_30_O_16_	Theaflavin-3-*O*-(3-*O*-methyl) gallate	1.31^d^	0.55^h^	2.20^a^	0.12^k^	0.61^g^	0.45^j^	1.39^c^	0.48^i^	1.60^b^	1.00^f^	1.27^e^
43	11.235	C_45_H_38_O_18_	Procyanidin C isomer 4	2.28^c^	0.77^h^	3.17^a^	1.40^e^	0.95^g^	0.47^i^	1.57^d^	1.16^f^	0.95^g^	1.00^g^	2.44^b^
44	11.492	C_34_H_26_O_22_	Sanguiin H1	2.58^f^	1.75^h^	15.40^a^	4.74^d^	3.85^e^	1.46^i^	2.36^g^	1.77^h^	5.12^c^	1.00^i^	6.41^b^
45	11.583	C_37_H_30_O_16_	Ent-epicatechin-(4α→ 8)-ent-epicatechin-3″-gallate	3.32^c^	3.47^b^	1.68^e^	0.67^h^	0.64^h^	0.16^i^	1.62^e^	4.27^a^	1.21^f^	1.00^g^	2.13^d^
46	11.791	C_44_H_34_O_21_	GCG-(4β→ 6)-ECG	2.16^b^	0.40^h^	5.90^a^	0.11^i^	1.26^d^	0.63^g^	1.64^c^	0.39^h^	0.58^g^	1.00^e^	0.90^f^
47	11.899	C_37_H_30_O_16_	Ent-epicatechin-(4α→ 8)-ent-epicatechin-3-gallate	2.54^b^	1.00^g^	8.04^a^	1.54^f^	0.60^i^	0.26^j^	1.84^e^	2.05^d^	0.68^h^	1.00^g^	2.31^c^
48	12.073	C_44_H_34_O_21_	ECG-(4β→ 8)-ECG	2.40^b^	0.56^g^	3.63^a^	0.08^h^	0.98^d^	0.56^g^	1.75^c^	0.60^f^	0.67^e^	1.00^d^	0.70^e^
49	14.140	C_44_H_34_O_21_	ECG-(4β→ 6)-ECG	1.46^c^	0.00^i^	3.18^b^	0.15^h^	1.08^d^	0.88^fg^	13.53^a^	0.00^i^	0.90^f^	1.00^e^	0.83^fg^
50	14.596	C_27_H_30_O_14_	Camellianin B	0.57^e^	2.00^b^	0.33^i^	0.40^h^	0.52^f^	0.95^c^	0.36^hi^	5.52^a^	0.46^g^	1.00^c^	0.66^d^
51	21.561	C_29_H_24_O_12_	Isoneotheaflavin	1.53^a^	0.00^h^	0.82^e^	0.11^h^	0.76^f^	0.57^g^	0.96^d^	1.33^b^	0.95^de^	1.00^cd^	1.10^c^
52	23.287	C_29_H_24_O_12_	Isotheaflavin	1.13^bc^	0.00^i^	0.95^ef^	0.00^i^	1.68^a^	0.87^f^	0.74^g^	0.30^h^	1.22^b^	1.00^de^	1.08^cd^
53	24.607	C_29_H_24_O_12_	Theaflavin	0.69^c^	0.20^d^	1.15^b^	0.18^d^	0.94^bc^	0.83^bc^	0.88^bc^	1.01^bc^	0.92^bc^	1.00^bc^	1.23^a^
54	24.865	C_36_H_28_O_16_	Neotheaflavin 3-*O*-gallate	0.89^bc^	0.21^f^	0.81^cd^	0.06^g^	0.92^b^	0.87^bc^	1.06^a^	0.57^e^	0.73^d^	1.00^ab^	0.63^e^
55	24.956	C_43_H_32_O_20_	Theaflavin-3,3″-digallate	1.52^b^	0.30^h^	0.80^e^	0.04^i^	1.05^cd^	1.09^c^	1.91^a^	0.44^g^	0.76^f^	1.00^de^	0.35^h^
56	24.981	C_36_H_28_O_16_	Theaflavin-3-gallate	0.82^de^	0.26^f^	0.99^a^	0.07^g^	0.78^e^	0.82^de^	0.94^b^	0.88^c^	0.88^c^	1.00^a^	0.83^d^
**Flavonols, flavones, and their glycosides**														
57	8.279	C15H12O	Chalcone	1.01bc	0.22f	1.12a	0.12g	0.97bc	0.65d	0.92c	0.49e	0.96c	1.00bc	1.06ab
58	10.355	C_27_H_30_O_15_	Isovitexin-2″-*O*-glucoside	0.69^cd^	0.89^b^	0.33^e^	0.05^f^	0.78^bc^	1.68^a^	0.30^ef^	0.95^b^	0.18^ef^	1.00^b^	0.55^d^
59	12.413	C_27_H_30_O_17_	Myricetin 3-*O*-rutinoside	0.62^e^	0.74^e^	1.12^d^	0.74^e^	0.68^e^	0.64^e^	1.73^c^	5.97^a^	1.88^c^	1.00^d^	2.93^b^
60	12.463	C_26_H_28_O_14_	Apigenin-6-*C*-glucoside-8-*C*-arabinoside (Isoschaftoside)^†^	1.52^f^	2.53^d^	1.23^i^	1.30^h^	3.40^c^	6.34^a^	1.40^g^	5.12^b^	1.02^j^	1.00^j^	2.32^e^
61	12.496	C_21_H_20_O_13_	Myricitin-3-*O*-galactoside^†^	0.40^bc^	0.12^c^	0.93^a^	0.13^c^	0.31^c^	0.25^c^	0.79^ab^	0.59^b^	0.15^c^	1.00^a^	0.54^bc^
62	12.770	C_21_H_20_O_13_	Myricitin-3-*O*-glucoside^†^	1.00^g^	1.65^d^	1.17^g^	0.43^h^	1.44^f^	1.51^ef^	1.80^c^	3.03^a^	2.04^b^	1.00^g^	1.59^de^
63	13.301	C_33_H_40_O_21_	Quercetin-3-*O*-glucosyl-rhamnosyl-galactoside	0.39^d^	0.18^f^	0.43^c^	0.05^gh^	0.20^f^	0.30^e^	0.29^e^	0.65^b^	0.02^h^	1.00^a^	0.06^g^
64	13.791	C_27_H_30_O_15_	Vitexin-2″-*O*-glucoside	1.03^e^	5.20^b^	0.75^f^	1.10^de^	1.00^e^	2.29^c^	1.27^d^	20.50^a^	0.35^g^	1.00^e^	1.17^de^
65	13.891	C_33_H_40_O_21_	Quercetin-3-*O*-glucosyl-rhamnosyl-glucoside	1.81^e^	1.89^e^	1.18^f^	0.41^g^	3.11^d^	3.69^c^	3.26^d^	7.80^a^	1.87^e^	1.00^f^	6.68^b^
66	13.891	C_21_H_20_O_12_	Quercetin-3-*O*-galactoside^†^	1.76^g^	1.86^f^	1.14^h^	0.43^j^	2.94^e^	3.51^c^	3.02^d^	7.58^a^	1.90^f^	1.00^i^	6.36^b^
67	14.289	C_33_H_40_O_20_	Quercetin-3-*O*-dirhamnosylgalactoside	0.00^c^	0.00^c^	4.03^a^	0.30^c^	0.00^c^	0.00^c^	0.00^c^	0.00^c^	0.00^c^	1.00^b^	0.00^c^
68	14.663	C_21_H_20_O_10_	Apigenin-8-*C*-glucoside (Vitexin)^†^	1.34^b^	2.50^a^	0.27^e^	0.21^e^	0.99^bc^	2.54^a^	0.56^de^	2.31^a^	1.19^bc^	1.00^bc^	0.79^cd^
69	14.858	C_21_H_20_O_12_	Quercetin-3-*O*-glucoside^†^	1.42^h^	9.30^b^	3.17^f^	8.58^c^	1.52^h^	2.26^g^	2.17^g^	40.41^a^	3.41^e^	1.00^i^	5.89^d^
70	14.895	C_27_H_30_O_16_	Rutin^†^	1.82^h^	11.12^b^	3.86^e^	10.26^c^	2.00^h^	2.81^f^	2.42^g^	459.16^a^	3.86^e^	1.00^i^	6.82^d^
71	15.003	C_27_H_30_O_14_	Kaempferol-di-*O*-rhamnoside	0.55^de^	2.57^b^	0.28^f^	0.60^d^	0.45^e^	1.07^c^	0.31^f^	7.17^a^	0.30^f^	1.00^c^	0.51^de^
72	15.019	C_33_H_40_O_20_	Kaempferol-3-*O*-galactosyl-rutinoside	0.50^b^	0.06^e^	0.19^d^	0.02^e^	0.20^d^	0.19^d^	0.27^c^	0.22^cd^	0.02^e^	1.00^a^	0.03^e^
73	15.070	C_21_H_20_O_10_	Apigenin-6-*C*-glucoside (Isovitexin)^†^	1.43^b^	1.24^c^	0.26^f^	0.13^g^	0.90^d^	2.38^a^	0.61^e^	2.42^a^	1.18^c^	1.00^d^	0.68^e^
74	16.246	C_33_H_40_O_19_	Kaempferol-7-*O*-rhamnosyl-rutinoside	0.04^de^	0.00^e^	2.84^a^	0.11^d^	0.01^de^	0.00^e^	0.03^de^	0.36^c^	0.38^c^	1.00^b^	0.04^de^
75	16.298	C_33_H_40_O_20_	Kaempferol-3-*O*-glucosyl-rutinoside	3.09^b^	0.41^i^	0.62^h^	0.05^j^	3.47^a^	3.10^b^	2.67^c^	1.07^f^	1.30^e^	1.00^g^	2.53^d^
76	16.315	C_27_H_30_O_15_	Kaempferol-3-*O*-rutinoside	2.70^b^	0.38^e^	5.70^a^	0.11^e^	2.89^b^	2.69^b^	2.20^c^	0.95^d^	0.32^e^	1.00^d^	1.91^c^
77	17.336	C_21_H_20_O_11_	Kaempferol-3-*O*-galactoside^†^	0.53^b^	0.14^bc^	1.04^a^	0.07^c^	0.21^bc^	0.18^bc^	0.33^bc^	0.17^bc^	0.34^bc^	1.00^a^	0.15^bc^
78	17.676	C_27_H_30_O_15_	Kaempferol-3-robinobioside	10.51^b^	3.38^d^	17.07^a^	2.86^e^	7.16^c^	6.32^c^	6.73^c^	6.78^c^	3.32^d^	1.00^f^	3.09^d^
79	17.684	C_21_H_20_O_11_	Kaempferol-3-*O*-glucoside^†^	1.84^e^	2.91^d^	1.75^e^	2.34^de^	3.64^c^	4.32^b^	2.87^d^	5.52^a^	2.79^d^	1.00^f^	2.75^d^
80	24.682	C_15_H_10_O_6_	Kaempferol^†^	2.95^b^	2.24^c^	3.13^b^	0.19^f^	1.73^d^	4.19^a^	1.75^d^	0.87^e^	1.45^d^	1.00^e^	0.00^f^
81	24.910	C_30_H_26_O_13_	Tiliroside	2.89^d^	0.00^h^	4.05^c^	0.00^h^	1.02^g^	1.24^f^	11.01^a^	0.93^g^	2.23^e^	1.00^g^	8.30^b^
**Phenolic acids**														
82	1.083	C_7_H_12_O_6_	Quinic acid^†^	1.21^a^	0.07^f^	0.62^e^	0.06^f^	0.69^d^	0.64^e^	1.02^c^	0.06^f^	1.16^b^	1.00^c^	1.02^c^
83	4.195	C_14_H_16_O_10_	Theogallin	1.67^b^	0.05^i^	0.91^e^	0.02^i^	1.01^d^	0.37^g^	1.45^c^	0.16^h^	1.79^a^	1.00^d^	0.84^f^
84	4.204	C_7_H_6_O_5_	Gallic acid^§,†^	1.31^c^	0.14^h^	0.03^i^	1.48^a^	1.38^b^	1.45^a^	1.03^d^	0.21^g^	0.45^f^	1.00^d^	0.76^e^
85	8.338	C_16_H_18_O_9_	3-*O*-Caffeoylquinic acid	0.35^e^	0.00^g^	1.71^b^	0.06^fg^	0.09^fg^	0.00^g^	0.22^ef^	0.12^fg^	58.79^a^	1.00^c^	0.75^d^
86	8.429	C_27_H_24_O_18_	1,2,6-Trigalloylglucose	0.87^e^	0.11^g^	0.57^f^	0.00^h^	1.10^b^	1.30^a^	0.93^d^	0.84^e^	0.91^de^	1.00^c^	0.56^f^
87	8.861	C_27_H_24_O_18_	1,2,4- Trigalloylglucose	33.31^b^	4.64^i^	59.66^a^	5.19^h^	21.50^c^	21.49^c^	17.23^f^	13.31^g^	19.12^e^	1.00^j^	20.3^d^
88	9.051	C_16_H_18_O_8_	3-*p*-Coumaroylquinic acid	1.40^a^	0.05^h^	0.60^d^	0.02^h^	0.32^e^	0.18^f^	0.25^f^	0.01^h^	0.96^c^	1.00^c^	1.18^b^
89	9.292	C_16_H_18_O_9_	5-*O*-Caffeoylquinic acid	1.43^d^	0.03^g^	6.00^a^	0.06^g^	1.45^d^	0.64^f^	3.15^c^	0.05^g^	5.80^b^	1.00^e^	1.46^d^
90	9.516	C_16_H_18_O_9_	4-*O*-Caffeoylquinic acid	0.20^f^	0.00^h^	3.29^a^	0.08^g^	0.14^f^	0.09^g^	0.81^d^	0.09^g^	3.15^b^	1.00^c^	0.32^e^
91	10.346	C_16_H_18_O_8_	4-*p*-Coumaroylquinic acid	1.56^a^	0.04^g^	1.16^b^	0.02^g^	1.07^bc^	0.50^e^	0.36^f^	0.12^g^	0.89^d^	1.00^cd^	1.59^a^
92	10.878	C_16_H_18_O_8_	5-*p*-Coumaroylquinic acid	1.72^a^	0.03^g^	1.13^c^	0.03^g^	0.99^d^	0.41^f^	0.65^e^	0.07^g^	1.13^c^	1.00^d^	1.46^b^
93	12.247	C_14_H_6_O_8_	Ellagic acid^†^	0.00^f^	0.00^f^	0.78^c^	0.00^f^	0.72^c^	0.36^d^	1.45^a^	0.00^f^	1.40^a^	1.00^b^	0.48^b^
**Amino acids**														
94	0.947	C_3_H_7_NO_3_	Serine	0.78^cd^	0.21^f^	0.45^e^	0.17^f^	0.77^cd^	1.00^b^	0.70^d^	0.49^e^	0.86^c^	1.00^b^	1.30^a^
95	0.958	C_4_H_7_NO_4_	Aspartic acid	0.63^h^	0.34^i^	0.35^i^	0.21^j^	2.04^b^	1.77^c^	1.12^e^	0.77^g^	1.48^d^	1.00^f^	2.55^a^
96	0.983	C_5_H_9_NO_4_	Glutamic acid	1.28^e^	0.61^i^	0.71^h^	0.41^j^	2.36^a^	2.11^b^	1.82^c^	0.94^g^	1.71^d^	1.00^f^	2.37^a^
97	1.523	C_7_H_14_N_2_O_3_	Theanine^†^	1.59^e^	0.35^i^	1.38^f^	0.20^j^	2.78^a^	2.46^b^	1.70^d^	0.63^h^	2.17^c^	1.00^g^	1.79^d^
98	2.070	C_9_H_11_NO_3_	Tyrosine	0.53^d^	0.10^h^	0.46^e^	0.02^i^	0.72^c^	0.30^f^	0.84^b^	0.16^g^	0.81^b^	1.00^a^	0.11^h^
**Alkaloids**														
99	7.770	C_7_H_8_N_4_O_2_	Theobromine^§,†^	7.21^a^	0.39^g^	2.36^b^	0.07^i^	1.32^e^	0.36^g^	2.19^c^	0.22^h^	1.54^d^	1.00^f^	0.37^g^
100	9.591	C_8_H_10_N_4_O_2_	Caffeine^§,†^	0.74^e^	0.12^g^	1.17^a^	0.04^h^	0.96^d^	1.00^d^	1.10^b^	0.20^f^	1.06^c^	1.00^d^	0.97^d^
**Organic acids**														
101	1.689	C_6_H_8_O_7_	Citric acid	1.77^b^	0.45^e^	0.91^d^	0.04^f^	1.73^b^	1.84^b^	1.27^c^	0.15^f^	1.84^b^	1.00^d^	3.65^a^
**Nucleosides**														
102	0.950	C_9_H_19_O_11_P	1-(sn-Glycero-3-phoshpo)-1D-myo-inositol	0.92^d^	0.20^j^	2.68^a^	0.39^i^	0.77^f^	0.69^g^	1.56^b^	0.08^k^	0.62^h^	1.00^c^	0.89^e^
103	1.062	C_10_H_12_N_4_O_5_	Inosine	0.97^c^	0.59^d^	1.39^b^	0.48^e^	1.39^b^	1.00^c^	2.77^a^	0.26^f^	1.09^c^	1.00^c^	1.35^b^
104	1.132	C_9_H_13_N_2_O_9_P	Uridine 2′-phosphate	7.72^d^	0.00^i^	3.14^g^	0.00^i^	29.93^a^	12.23^b^	8.74^c^	0.00^i^	5.89^e^	1.00^h^	3.97^f^
105	1.390	C_9_H_13_N_2_O_9_P	3′-UMP	1.57^d^	0.03^g^	2.21^b^	0.02^g^	2.56^a^	1.66^c^	2.18^b^	0.52^f^	1.01^e^	1.00^e^	2.20^b^
106	2.842	C_9_H_13_N_2_O_9_P	Uridine 5′-monophosphate	0.18^h^	0.00^i^	0.58^f^	0.00^i^	0.66^e^	0.73^d^	0.33^g^	0.79^c^	0.80^c^	1.00^a^	0.95^b^
107	4.204	C_9_H_13_N_2_O_9_P	Pseudouridine 5′-phosphate	2.79^c^	0.00^i^	1.56^d^	0.00^e^	0.76^g^	1.43^e^	2.90^b^	1.58^d^	4.02^a^	1.00^f^	0.58^h^
**Carbohydrates**														
108	0.991	C_6_H_12_O_7_	Gluconic acid	0.96^d^	0.33^g^	0.21^h^	0.14^i^	0.91^e^	0.19^i^	0.19^e^	0.86^f^	1.76^a^	1.00^c^	1.25^b^
109	1.004	C_18_H_32_O_16_	Maltotriose	1.45^i^	16.05^a^	3.06^f^	4.77^c^	2.48^g^	3.22^e^	4.38^d^	13.18^b^	1.31^j^	1.00^k^	1.77^h^
110	1.083	C_12_H_22_O_11_	Maltose	1.28^d^	3.06^a^	0.86^ef^	2.27^b^	0.96^e^	1.74^c^	0.67^g^	1.60^c^	0.76^fg^	1.00^e^	0.80^fg^

*The anthocyanins identified in the present study were not shown in this table; data were assessed by one-way ANOVA followed by Fisher’s LSD test; values in the same row that are labeled with different letters (a–k) differ significantly (*p* < 0.05). ^#^The letters A and B after tea cultivar name represent the new leaves (one bud and two leaves) and old leaves, respectively. ^†^The compounds were identified by authentic standards. ^§^Relative fold of the compounds was calculated according to the data of HPLC-UV.

#### Anthocyanin Identification and Measurement in Purple-Leaf Tea Cultivars

Seven main chromatographic peaks were detected in NL-PTC, representing at least seven anthocyanins. However, only one small chromatographic peak was detected in OL-PTC, NL-GTC, and NL-YTC. This indicated that only NL-PTC were rich in anthocyanins, which was consistent with their leaf color. In order to identify the aglycone form of anthocyanins, the tea leaf extracts were first subjected to acid hydrolysis, then the reaction solution was separated and detected by the UPLC-DAD-QTOF-MS system. Three aglycones including delphinidin, cyanidin, and pelargonidin were validated by UV-Vis spectra and MS and MS/MS characteristic spectral data; the results were further confirmed by co-elution with the corresponding standards (Deng et al., 2013).

The anthocyanins corresponding to the seven peaks were further identified using the QTOF-MS system at PI mode. Peak 1 showed a molecular ion [M]^+^ at 465.1054, which was calculated as a molecular formula of C_21_H_21_O_12_^+^ (*m/z* 465.1033 for its theoretical mass). The MS^2^ spectrum exhibited a major fragment ion at *m/z* 303.0524 corresponding to the aglycon delphinidin (C_15_H_11_O_7_^+^; *m/z* 303.0505), which was due to the loss of a hexose moiety (C_6_H_10_O_5_). Peak 1 was tentatively assigned to delphinidin-3-*O*-hexose, then further confirmed as delphinidin-3-*O*-galactoside by co-elution with the corresponding standard. Peaks 5 and 6 yielded molecular ions [M]^+^ at 611.1438 and 611.1429, respectively, which agreed well with the calculated mass of C_30_H_27_O_14_^+^ (*m/z* 611.1401). Similar to peak 1, peaks 5 and 6 had a major fragment ion at m/z 303.0542 and 303.0518, respectively, indicating that they were also delphinidin glycosides. The formation of a delphinidin ion in the MS^2^ spectrum may be attributed to loss of a coumaroyl moiety and a hexose moiety (C_9_H_6_O_2 +_ C_6_H_10_O_5_). Peaks 5 and 6 were identified as delphinidin-(*Z*)-*p*-coumaroylgalactoside and delphinidin-(*E*)-*p*-coumaroylgalactoside, respectively, through a comparison with published data (Saito et al., 2011; Maeda-Yamamoto et al., 2012; Jiang et al., 2013). For peaks 2 and 7, fragment ions were observed at *m/z* 287.0596 and 287.0586, corresponding to the aglycon cyanidin (C_15_H_11_O_6_^+^; *m/z* 287.0556 for its theoretical mass), with their molecular ions [M]^+^ at *m/z* 449.1102 and 595.1466, corresponding to the molecular formula of C_21_H_21_O_11_^+^ (*m/z* 449.1084) and C_30_H_27_O_13_^+^ (*m/z* 595.1452), respectively. Peaks 2 and 7 were assigned as cyanidin glycosides, and the formation of their cyanidin ion in the MS^2^ spectrum was due to the loss of a hexose moiety and a coumaroyl + hexose moiety, respectively. Peak 7 was identified as cyanidin-(*E*)-*p*-coumaroylgalactoside (Saito et al., 2011; Maeda-Yamamoto et al., 2012; Jiang et al., 2013). Peak 2 was assigned to cyanidin-3-*O*-galactoside, which was confirmed by co-elution with the standard. In addition, peaks 3 and 4 showed molecular ions [M]^+^ at 443.1158 and 595.1698, corresponding to the molecular formula of C_21_H_21_O_10_^+^ (*m/z* 433.1135) and C_27_H_31_O_15_^+^ (*m/z* 595.1663), respectively, with their major fragment ions at *m/z* 271.0634 and 271.0644, corresponding to the aglycon pelargonidin (C_15_H_11_O_5_^+^; *m/z* 271.0606). The formation of a pelargonidin ion at peaks 3 and 4 was due to the loss of one hexose moiety and two hexose moieties, respectively. Peaks 3 and 4 were tentatively identified as pelargonidin-*O*-hexose and pelargonidin-*O*-dihexose (Lu et al., 2015).

The anthocyanins were quantified using the calibration curve of cyanidin-3-*O*-galactoside at 520 nm. As shown in Figures 1C,D, the anthocyanin composition and content varied widely among the NL-PTC, OL-PTC, NL-GTC, and NL-YTC. NL-PTC was rich in anthocyanins, with an anthocyanin content ranging from 243 μg/g of DW to 1173 μg/g of DW. Further, the anthocyanin content in the new leaves of dark purple-leaf tea cultivars (HYC-A and ZJ-A) was higher than that in the new leaves of light purple-leaf tea cultivars (9803-A and HYFS-A). Delphinidin glycosides and cyanidin glycosides were the major anthocyanins in NL-PTC, with a concentration of 121–573 μg/g of DW (48.8–65.7% of total anthocyanins) and 60–564 μg/g of DW (19.7–48.1% of total anthocyanins), respectively. A low content of pelargonidin glycosides (36–63 μg/g of DW) was observed in NL-PTC. Furthermore, delphinidin-(*E*)-*p*-coumaroylgalactoside and cyanidin-(*E*)-*p*-coumaroylgalactoside accounted for the two highest levels of anthocyanins in NL-PTC, with concentrations of 83–410 μg/g of DW and 38–434 μg/g of DW, respectively. However, except for 9803-B, which had a relatively high level of anthocyanins (113 μg/g of DW), no anthocyanins or only a low concentration of pelargonidin-*O*-dihexose or cyanidin-3-*O*-galactoside were found in OL-PTC, NL-GTC, and NL-YTC, with the total anthocyanin concentration ranging from 0 to 41 μg/g of DW. Further, the total anthocyanin content in 9803-B was also significantly lower than that in NL-PTC.

#### Other Types of Flavonoid in Purple-Leaf Tea Cultivars

**FIGURE 3:**
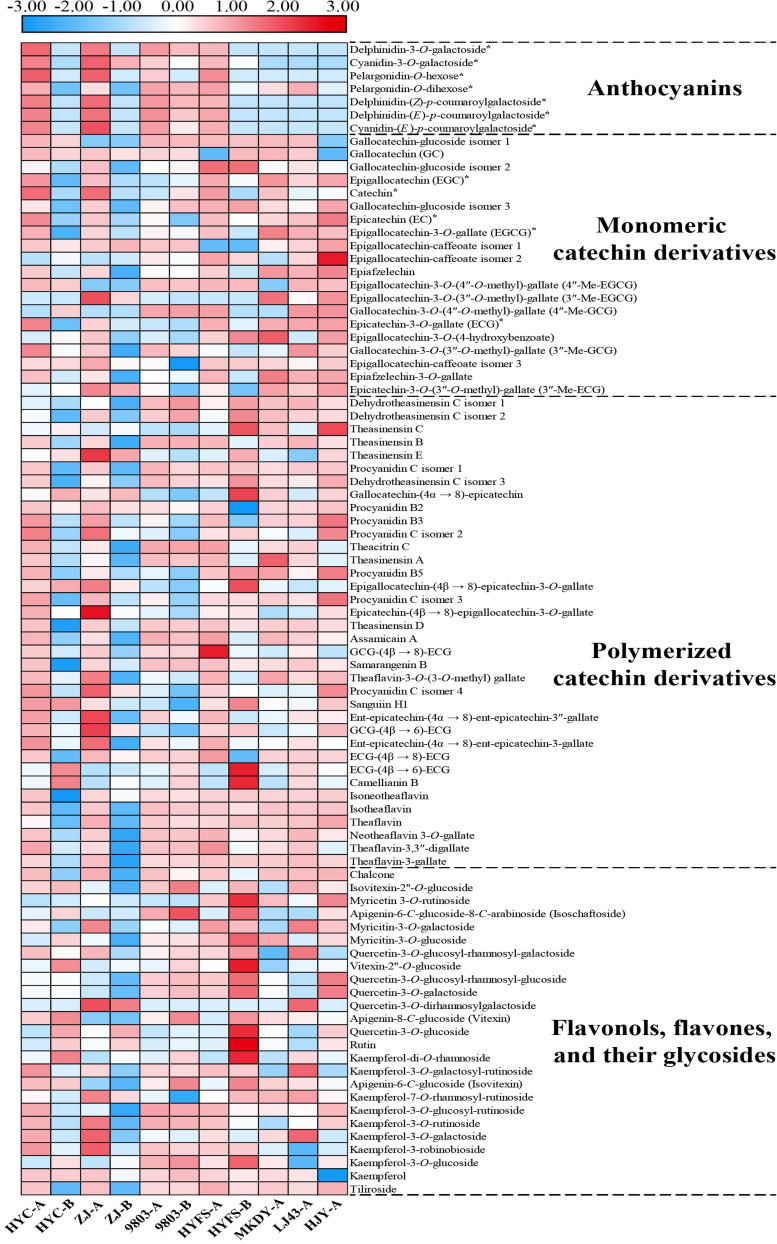
Heat-map of anthocyanin/flavonoid content in the leaves of purple-leaf, green-leaf, and yellow-leaf tea cultivars according to UPLC-QTOF-MS and HPLC-UV data. *Heat-map of the compound was obtained from HPLC-UV chromatogram data. The letters A and B after the tea cultivar name represent new leaves (one bud and two leaves) and old leaves, respectively.

Other types of flavonoid, including monomeric catechin derivatives, polymerized catechin derivatives, flavonols, and flavones, were also identified and profiled according to the method of a previous study (Table 1 and Figure 3; Zhu et al., 2017). The levels of six major monomeric catechin derivatives, including EGCG, ECG, EGC, GCG, EC, and catechin, were also measured by HPLC-UV (Table 2; Gong et al., 2020). The EGCG content in NL-PTC ranged from 17.49 to 37.54 mg/g of DW, which was higher than that in OL-PTC (0.96–13.33 mg/g of DW). Further, the total amount of six monomeric catechin derivatives in NL-PTC (29.20–92.64 mg/g of DW) was also higher than that in OL-PTC (3.78–22.21 mg/g of DW). In addition, both the EGCG content and total content of six monomeric catechin derivatives in the new leaves of green-leaf CSS (NL-G-CSS) (LJ43-A) and the new leaves of purple-leaf CSS (NL-P-CSS) (HYC-A, 9803-A, and HYFS-A) were lower than those in the new leaves of green-leaf CSA (NL-G-CSA) (MKDY-A) and the new leaves of purple-leaf CSA (NL-P-CSA), respectively. Moreover, the EGCG content and total content of six monomeric catechin derivatives in NL-P-CSA were 41.5–72.7% and 8.6–71.2% lower than those in NL-G-CSA, respectively. Furthermore, both the EGCG content and total content of six monomeric catechin derivatives in NL-P-CSS were 16.2–53.3% and 1.5–58.4% lower than those in NL-G-CSS, respectively; however, no difference was observed between HYFS-A and LJ43-A regarding the total content of six monomeric catechin derivatives. Moreover, the content of some other monomeric catechin derivatives (e.g., epiafzelechin and epiafzelechin-3-*O*-gallate) in NL-PTC was also lower than that in NL-GTC (Table 1).

**TABLE 2:** Major chemical constituents in the leaves of purple-leaf, green-leaf, and yellow-leaf tea cultivars measured by HPLC-UV^a^.

Chemical constituents	HYC-A	HYC-B	ZJ-A	ZJ-B	9803-A	9803-B	HYFS-A	HYFS-B	MKDY-A	LJ43-A	HJY-A
EGCG (mg/g)	25.14 ± 0.97^d^	6.41 ± 0.07^g^	37.54 ± 0.64^b^	0.96 ± 0.05^h^	17.49 ± 0.14^e^	13.33 ± 0.81^f^	31.39 ± 0.13^c^	7.54 ± 0.17^g^	64.18 ± 1.66^a^	37.47 ± 1.70^b^	32.33 ± 0.88^c^
ECG (mg/g)	9.06 ± 0.29^f^	2.94 ± 0.01^gh^	21.34 ± 0.66^a^	0.26 ± 0.01^j^	2.42 ± 0.03^h^	1.65 ± 0.10^i^	13.02 ± 0.08^e^	3.51 ± 0.11^g^	13.61 ± 0.54^d^	14.33 ± 0.64^c^	15.19 ± 0.03^b^
EGC (mg/g)	8.78 ± 0.68^c^	2.01 ± 0.01^g^	12.54 ± 0.13^a^	0.56 ± 0.08^h^	2.43 ± 0.16^f^	3.81 ± 0.14^e^	10.60 ± 0.12^b^	4.54 ± 0.15^d^	12.89 ± 0.44^a^	7.26 ± 0.59^c^	10.92 ± 0.09^b^
GCG (mg/g)	0.68 ± 0.04^ab^	0.13 ± 0.00^de^	0.70 ± 0.03^a^	0.15 ± 0.01^de^	0.47 ± 0.08^c^	0.61 ± 0.04^b^	0.76 ± 0.08^a^	0.08 ± 0.01^e^	0.20 ± 0.01^d^	0.47 ± 0.08^c^	0.18 ± 0.04^d^
EC (mg/g)	9.12 ± 0.34^d^	1.84 ± 0.06^g^	15.88 ± 0.11^b^	1.48 ± 0.08^gh^	5.76 ± 0.11^f^	1.05 ± 0.06^h^	10.05 ± 0.78^c^	5.29 ± 0.30^f^	8.08 ± 0.09^e^	9.64 ± 0.28^c^	18.28 ± 0.06^a^
Catechin (mg/g)	4.60 ± 0.02^a^	0.48 ± 0.35^gh^	4.62 ± 0.06^a^	0.36 ± 0.02^h^	0.64 ± 0.08^g^	1.76 ± 0.07^d^	3.28 ± 0.02^b^	0.36 ± 0.09^h^	2.39 ± 0.22^c^	1.00 ± 0.02^f^	1.28 ± 0.08^e^
Total catechin derivatives^b^ (mg/g)	57.37 ± 0.42^e^	13.81 ± 0.48^h^	92.64 ± 1.64^b^	3.78 ± 0.02^i^	29.20 ± 0.60^f^	22.21 ± 1.14^g^	69.09 ± 0.61^d^	21.33 ± 0.02^g^	101.34 ± 2.05^a^	70.17 ± 0.70^d^	78.18 ± 0.59^c^
Gallic acid (mg/g)	0.38 ± 0.01^c^	0.04 ± 0.01^h^	0.01 ± 0.01^i^	0.43 ± 0.01^a^	0.40 ± 0.01^b^	0.42 ± 0.01^a^	0.30 ± 0.02^d^	0.06 ± 0.00^g^	0.13 ± 0.00^f^	0.29 ± 0.01^d^	0.22 ± 0.01^e^
Caffeine (mg/g)	17.66 ± 0.39^e^	2.94 ± 0.17^g^	27.74 ± 0.07^a^	0.87 ± 0.01^h^	22.73 ± 0.22^d^	23.70 ± 0.20^d^	26.21 ± 1.34^b^	4.72 ± 0.33^f^	25.19 ± 1.60^c^	23.72 ± 0.14^d^	22.92 ± 0.06^d^
Theobromine (mg/g)	20.05 ± 0.43^a^	1.08 ± 0.10^g^	6.56 ± 0.08^b^	0.20 ± 0.02^i^	3.67 ± 0.08^e^	1.01 ± 0.08^g^	6.10 ± 0.46^c^	0.60 ± 0.11^h^	4.28 ± 0.04^d^	2.78 ± 0.09^f^	1.03 ± 0.06^g^
Theophylline (mg/g)	0.00 ± 0.00^c^	0.00 ± 0.00^c^	0.07 ± 0.03^a^	0.00 ± 0.00^c^	0.02 ± 0.01^b^	0.02 ± 0.00^b^	0.03 ± 0.01^b^	0.00 ± 0.00^c^	0.00 ± 0.00^c^	0.02 ± 0.00^b^	0.00 ± 0.00^c^
Total alkaloids^c^ (mg/g)	37.71 ± 0.04^a^	4.02 ± 0.07^i^	34.36 ± 0.02^b^	1.08 ± 0.01^j^	26.41 ± 0.32^f^	24.73 ± 0.13^g^	32.33 ± 0.87^c^	5.32 ± 0.22^g^	29.47 ± 1.55^d^	26.52 ± 0.23^e^	23.95 ± 0.04^g^

*^*a*^The letters A and B after tea cultivar name represent the new leaves (one bud and two leaves) and old leaves, respectively; values in the same row that are labeled with different letters (a–j) differ significantly (*p* < 0.05). Data were assessed by one-way ANOVA followed by Fisher’s LSD test. ^*b*^Total catechin derivatives including EGCG, ECG, EGC, GCG, EC, and catechin. ^*c*^Total alkaloids including caffeine, theobromine, and theophylline. EGCG, epigallocatechin-3-*O*-gallate; ECG, epicatechin-3-*O*-gallate; EGC, epigallocatechin; GCG, gallocatechin-3-*O*-gallate; EC, epicatechin.*

As shown in Table 1 and Figure 3, the levels of 10 out of 35 polymerized catechin derivatives in NL-P-CSS and NL-P-CSA were higher than those in the NL-G-CSS and NL-G-CSA. These 10 polymerized catechin derivatives were theasinensin E, procyanidin C isomers 1 and 2, epicatechin-(4β→ 8)-epigallocatechin-3-*O*-gallate, theasinensin D, GCG-(4β→ 8)-ECG, samarangenin B, Sanguiin H1, GCG-(4β→ 6)-ECG, and ECG-(4β→ 6)-ECG. In addition, the levels of nine other polymerized catechin derivatives in NL-P-CSS and NL-P-CSA were also higher than those in NL-G-CSS and NL-G-CSA, except for 9803-A. These nine polymerized catechin derivatives were procyanidins B2 and B3, epigallocatechin-(4β→ 8)-epicatechin-3-*O*-gallate, theaflavin-3-*O*-(3-*O*-methyl) gallate, procyanidin C isomer 4, ent-epicatechin-(4α→ 8)-ent-epicatechin-3″-gallate, ent-epicatechin-(4α→ 8)-ent-epicatechin-3-gallate, ECG-(4β→ 8)-ECG, and theaflavin-3,3″-digallate. The isoschaftoside, kaempferol-3-robinobioside, and kaempferol contents in NL-P-CSS and NL-P-CSA were higher than those in NL-G-CSS and NL-G-CSA (Table 1 and Figure 3). Nevertheless, most flavonols and flavones identified in our study showed different trends in the CSS and CSA. The levels of isovitexin-2″-*O*-glucoside, myricitin-3-*O*-galactoside, quercetin-3-*O*-glucosyl-rhamnosyl-galactoside, kaempferol-3-*O*-galactosyl-rutinoside, kaempferol-7-*O*-rhamnosyl-rutinoside, kaempferol-3-*O*-rutinoside, and kaempferol-3-*O*-galactoside in NL-P-CSS were lower than those in NL-G-CSS, whereas the levels of these compounds in NL-P-CSA were higher than those in NL-G-CSA. In contrast, the levels of myricitin-3-*O*-glucoside, quercetin-3-*O*-glucosyl-rhamnosyl-glucoside, quercetin-3-*O*-galactoside, quercetin-3-*O*-glucoside, kaempferol-3-*O*-glucosyl-rutinoside, and kaempferol-3-*O*-glucoside in NL-P-CSS were higher than those in NL-G-CSS, whereas the levels of these compounds in NL-P-CSA were lower than those in NL-G-CSA.

### Biosynthesis of Anthocyanin/Flavonoid in Purple-Leaf Tea Cultivars

**FIGURE 4:**
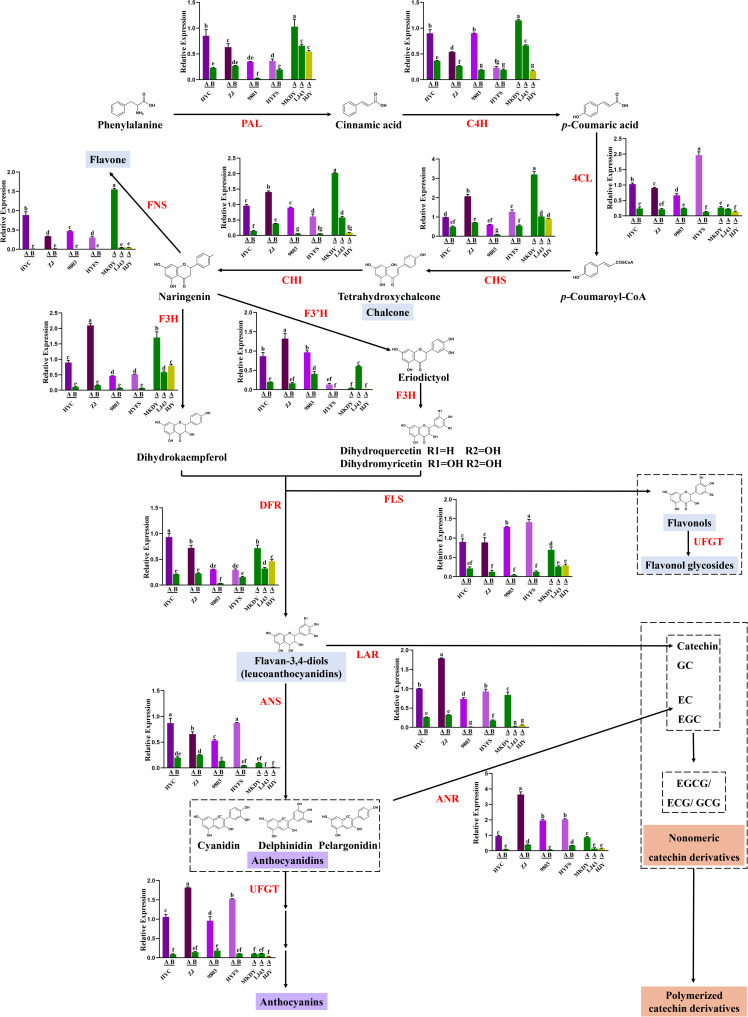
Changes of gene expression involving anthocyanin/flavonoid biosynthesis in the leaves of purple-leaf, green-leaf, and yellow-leaf tea cultivars. Values in the same row that are labeled with different letters (a–g) differ significantly (*p* < 0.05). PAL, phenylalanine ammonia lyase; C4H, cinnamate-4-hydroxylase; 4CL, 4-coumarate: CoA ligase; CHS, chalcone synthase; CHI, chalcone isomerase; FNS, flavone synthase; F3′H, flavonoid 3′-hydroxylase; F3H, flavanone-3-hydroxylase; FLS, flavonol synthase; DFR, dihydroflavonol 4-reductase; LAR, leucoanthocyanidin reductase; ANS, anthocyanidin synthase; ANR, anthocyanidin reductase, UFGT, UDP-glucose: flavonoid 3-*O*-glucosyltransferase.

The expressions of 14 structural genes in the anthocyanin/flavonoid biosynthetic pathway are shown in Figure 4. The 14 gene expressions of new leaves were higher than those of old leaves in the same purple-leaf tea cultivar, but no difference was observed in the expression of *C4H*, *F3*′*H*, and *DFR* genes between HYFS-A and HYFS-B. Besides, the expression of most of the 14 genes including *PAL*, *C4H*, *CHS*, *CHI*, *FNS*, *F3H*, *FLS*, *DFR*, *LAR*, and *ANR* in MKDY-A was higher than that in LJ43-A. Moreover, the expressions of *4CL*, *ANS*, *UFGT*, and *FLS* genes in NL-PTC were significantly higher than those in NL-GTC. Further, the expressions of *LAR* and *ANR* genes in NL-P-CSS and NL-P-CSA were higher than those in NL-G-CSS and NL-G-CSA, respectively.

## Discussion

Purple-leaf tea cultivars have drawn increasing research attention due to their specific chemical composition that greatly influences the tea quality and biological activity, as well as plant resistance (Lai et al., 2016; Kim et al., 2017; Zhou et al., 2017). Previous studies have confirmed that the purple leaf color of tea cultivars is closely associated with anthocyanin accumulation (Shen et al., 2018). However, the molecular mechanism underlying purple-leaf formation in tea cultivars remains unclear. In this study, we systematically studied the biosynthesis of three pigments (chlorophylls, carotenoids, and anthocyanins/flavonoids) in four NL-PTC by integrated metabolic and gene expression analyses, and elucidated the mechanism of purple-leaf formation in tea cultivars. We also studied the biosynthesis of these three pigments in OL-PTC, two NL-GTC, and one NL-YTC that served as a comparison.

### Chlorophyll Synthesis as a Mechanism of Purple-Leaf Formation in Tea Cultivars

Chlorophylls, which are the Mg^2+^-containing tetrapyrrole compounds essential for light harvesting and energy transduction in photosynthesis, are responsible for the green color in leaves (Beale, 2005). Previous studies have shown that low chlorophyll in the leaves of yellow-leaf tea cultivars is related to chloroplast degradation and chlorophyll metabolism (Wang et al., 2014). However, the biosynthesis of chlorophylls in purple-leaf tea cultivars remain unknown. According to our results, the chlorophyll a + b content in NL-PTC was lower than that in NL-GTC, indicating that the purple color formation in the new leaves of tea was related to a decrease in chlorophyll level, as well as high level of purple pigments. In addition, the level of chlorophyll a + b in NL-PTC was lower than that in OL-PTC, which was consistent with previous studies (Wang et al., 2017).

Chlorophyll biosynthesis is a complex process, which can be divided into three phases: (I) formation of δ-aminolevulinic acid (ALA) by conversion of glutamate, (II) formation of protoporphyrin IX, and (III) the Mg-protoporphyrin pathway producing chlorophylls. A series of genes are involved in the three phases of chlorophyll biosynthesis (Wang et al., 2014). We compared the expression patterns of nine key genes in chlorophyll biosynthesis and observed substantial differences. Specifically, the expression of the *HEML* gene in NL-PTC was lower than that in NL-GTC, but no difference was observed between HYFS-A and NL-GTC. The *HEME* gene is primarily responsible for the formation of ALA. The protein encoded by the *HEME* gene is functionally an aminomutase, which transfers the amino group from the carbon 2 of L-glutamate-1-semialdehyde to the carbon 5 of ALA. The *HEME* gene has been isolated from various plants, such as barley, tomato, soybean, and tobacco (Li et al., 2019). It was reported that the RNAi silenced expression of the *HEME* gene in tobacco resulted in a reduced content of chlorophyll and *HEME*, as well as reduced enzyme activities for Mg chelatase and Fe chelatase; however, no reduction occurred in the transcript levels of the genes encoding the subsequent steps in tetrapyrrole biosynthesis (Hedtke et al., 2007). Substantial differences in *HEME* expression may be the main reason for the variation of chlorophyll content between NL-PTC and NL-GTC. In addition, the *NOL* expression of new leaves was lower than or equal to that of old leaves in the same purple-leaf tea cultivar. The *NOL* gene is responsible for the conversion of chlorophyll b to chlorophyll a, thus leading to changes in the chlorophyll a/b ratio in leaves (Sato et al., 2015). The difference in *NOL* expression may be one reason for the variation of chlorophyll content between new leaves and old leaves in the same purple-leaf tea cultivar.

### Carotenoid Synthesis in Purple-Leaf Tea Cultivars

Carotenoids, the accessory light-harvesting pigments that trap and transfer light energy to chlorophylls, are responsible for the red, orange, and yellow color in tea leaves. Carotenoids are synthesized in plastids (e.g., chromoplasts and chloroplasts) by various enzymes (Zhao and Tao, 2015). Substantial progress has been made in the carotenoid accumulation of yellow-leaf tea cultivars. It was previously reported that the carotenoid content in NL-YTC is lower than that in NL-GTC (Song et al., 2017). The same result was obtained in this study. Moreover, we observed substantial variation of carotenoid content within the group of green-leaf tea cultivars. Further, expression of the *PSY* gene in MKDY-A was higher than that in LJ43-A. Carotenoid biosynthesis is also a complex process, in which the enzyme encoded by the *PSY* gene is the first rate-limiting regulatory enzyme. This enzyme catalyzes geranylgeranyl diphosphate to phytoene, which is a precursor of plastidial isoprenoids (e.g., carotenoids) (Song et al., 2017). Previous studies have proven that down-regulation of the *PSY* gene resulted in decreased carotenoid content and efficiency of photosynthetic electron transport in *Oncidium hybrid* orchids, as well as a reduced chlorophyll level and decreased expression of chlorophyll biosynthetic genes (Liu et al., 2014). The low expression of the *PSY* gene may be the main cause of the low level of carotenoids in LJ43-A relative to MKDY-A. Low expression of the *PSY* gene may also contribute to the low level of chlorophylls in LJ43-A relative to MKDY-A. Moreover, we found that the carotenoid content in NL-PTC was lower than that in MKDY-A, but higher than or equal to that in LJ43-A, except for in 9803-A. Further, the expression of the *PSY* gene in NL-PTC was lower than that in MKDY-A, although no difference was observed between ZJ-A and MKDY-A; the expression of the *PSY* gene in NL-PTC was higher than or equal to that in LJ43-A. Thus, we speculate that *PSY* may be the key gene for carotenoid biosynthesis in tea leaves, as well as an important factor affecting chlorophyll level in tea leaves.

### Anthocyanin/Flavonoid Synthesis as a Mechanism of Purple-Leaf Formation in Tea Cultivars

#### Anthocyanin Accumulation

High anthocyanin accumulation is considered to be the major cause of the purple-leaf phenotype in tea plants (Lai et al., 2016; Sun et al., 2016). Several studies have investigated the composition and content of anthocyanins in some purple-leaf tea cultivars, but their results varied wildly (Saito et al., 2011; Maeda-Yamamoto et al., 2012; Lai et al., 2016). For instance, Jiang et al. (2013) reported that the total amount of anthocyanins in the ZJ cultivar was 707 ± 28 μg/g of DW, whereas Lai et al. (2016) reported a total content of anthocyanins in the ZJ cultivar of 58.91–69.72 mg/100 g of fresh weight. There is a notable lack of systematic studies on the anthocyanins of multiple purple-leaf tea cultivars. Metabolic profiling based on the UPLC-DAD-QTOF-MS system was used in our study to reveal the composition of anthocyanins in the four NL-PTC, and the anthocyanin content was further measured by UPLC-DAD analysis. The four purple-leaf tea cultivars were HYC, ZJ, 9803, and HYFS; new leaves of the first two are dark purple, whereas new leaves of the second two are light purple. Therefore, our study covers varying degrees of purple leaves. Furthermore, as far as we know, the biosynthesis of anthocyanin/flavonoid in HYC, 9803, and HYFS were analyzed for the first time in this study.

We identified seven anthocyanins in the four NL-PTC. Delphinidin-(*E*)-*p*-coumaroylgalactoside and cyanidin-(*E*)-*p*-coumaroylgalactoside were the two highest levels of anthocyanins in NL-PTC; delphinidin glycosides and cyanidin glycosides were also the two major anthocyanins in NL-PTC, which was consistent with previous findings (Jiang et al., 2013). The anthocyanin content varied wildly in the four NL-PTC. Further, the new leaves of dark purple-leaf tea cultivars (HYC and ZJ) had a higher level of anthocyanins than the new leaves of light purple-leaf tea cultivars (9803 and HYFS). In contrast, either no anthocyanins or low levels of anthocyanins were found in OL-PTC, NL-GTC, and NL-YTC. This suggests that the anthocyanin content determines whether tea leaves appear purple, as well as the shade of purple.

#### Accumulation of Other Types of Flavonoids

Besides the six anthocyanins, a total of 81 other flavonoids were identified by metabolic profiling analysis, as well as 12 phenolic acids, five amino acids, two alkaloid, one organic acid, six nucleosides, and three carbohydrates (Zhu et al.,2015a,b, 2016, 2017, 2018). To the best of our knowledge, this is the first time that flavonoid level has been systematically investigated in NL-PTC, OL-PTC, NL-GTC, and NL-YTC. Our results showed that CSS had a lower amount of EGCG and a lower total amount of six monomeric catechin derivatives than CSA of the same leaf color, which was consistent with previous results (Wan, 2008). Thus, flavonoid level in CSS and CSA were analyzed separately.

The EGCG content and total content of six monomeric catechin derivatives in NL-P-CSA and NL-P-CSS were lower than those in NL-G-CSA and NL-G-CSS, respectively, although no difference was observed in the total content of six monomeric catechin derivatives between HYFS-A and LJ43-A. Further, the levels of some other monomeric catechin derivatives (e.g., epiafzelechin and epiafzelechin-3-*O*-gallate) in NL-PTC were also lower than those in NL-GTC. This indicated that the purple-leaf phenotype and the corresponding high anthocyanin level may result in a reduction of monomeric catechin derivative level in the tea leaves. In contrast, most of the polymerized catechin derivatives in NL-P-CSS and NL-P-CSA were higher than those in NL-G-CSS and NL-G-CSA, respectively.

#### Anthocyanin/Flavonoid Biosynthesis

Anthocyanin/flavonoid biosynthesis involves multiple structural genes. These genes include *PAL*, *C4H*, *4CL*, *CHS*, *CHI*, *FNS*, *F3H*, *F3*′*H*, *FLS*, *DFR*, *LAR, ANS*, *ANR*, and *UFGT*, of which the first nine are upstream genes and the last five are downstream genes (Shen et al., 2018). We found that most structural genes in anthocyanin/flavonoid biosynthesis were up-regulated in new leaves compared with old leaves in the same purple-leaf tea cultivar, which was consistent with previous studies (Li et al., 2017). The low expressions of structural genes in old tea leaves were presumed as the main cause of the low anthocyanin/flavonoid content in the old tea leaves. Further, the majority of structural genes were down-regulated in LJ43-A compared with MKDY-A, which was consistent with low flavonoid level in LJ43-A relative to MKDY-A. This indicated that high flavonoid level in CSA is attributed to high gene expression in the flavonoid biosynthesis relative to CSS. Moreover, the *4CL*, *ANS*, *UFGT*, and *FLS* genes were up-regulated in NL-PTC compared with NL-GTC. *4CL* is the upstream gene of anthocyanin/flavonoid biosynthesis that catalyzes *p*-coumaric acid to form *p*-coumaroyl CoA. Activation of the *4CL* gene is reportedly responsible for anthocyanin/flavonoid accumulation in various plants (Li et al., 2018b). *ANS* and *UFGT* are key genes in the late stage of anthocyanin biosynthesis. *ANS* gene catalyzes the conversion of leucoanthocyanidin to colored anthocyanidin using Fe^2+^ and 2-oxoglutarate, and the colored anthocyanidin is eventually converted into anthocyanins under catalysis of the *UFGT* gene. Numerous studies have proved that anthocyanin accumulation is predominantly controlled by downstream genes including *ANS* and *UFGT* in various plants, although other structural genes also contribute to anthocyanin accumulation (Shen et al., 2018). It is presumed that the up-regulated expression of *4CL*, *ANS*, and *UFGT* genes involving both upstream and downstream biosynthesis of anthocyanin could lead to increased anthocyanin accumulation, enabling production of the purple-leaf tea plant phenotype. In addition, the expressions of downstream *LAR* and *ANR* genes in NL-P-CSS and NL-P-CSA were higher than those in NL-G-CSS and NL-G-CSA, respectively. *LAR* and *ANR* are key genes for the formation of monomeric and polymerized catechin derivatives in tea leaves (Wei et al., 2018). The high expressions of *LAR* and *ANR* genes may be the main reason for the high levels of polymerized catechin derivatives in NL-PTC relative to NL-GTC.

Numerous studies have proved that the structural genes in anthocyanin/flavonoid biosynthesis, especially the downstream genes including *ANS* and *UFGT*, are regulated by the transcription factors in a spatial-temporal pattern. These transcription factors include MYB, bHLH, and WD40 (Zhao and Tao, 2015; Wang et al., 2018). Sun et al. (2016) reported that activation of the R2R3-MYB transcription factor specifically up-regulated the bHLH transcription factor CsGL3 and recruited the WD-repeat protein CsTTG1 to form the MYB-bHLH-WDR complex, leading to the up-regulated expression of anthocyanin late biosynthetic genes and the subsequent ectopic accumulation of pigments in purple tea. In addition, it has been reported that the post-transcriptional regulation (e.g., miRNA and lncRNA regulation) plays a critical role in the regulation of anthocyanin/flavonoid biosynthesis of tea plants. Research on the regulation of anthocyanin/flavonoid biosynthesis has recently focused on post-transcriptional regulation (Wei et al., 2018; Zhao et al., 2019). Therefore, future studies should focus on the transcription factor regulatory and post-transcriptional regulation of anthocyanin/flavonoid biosynthesis during purple-leaf formation in tea cultivars.

## Conclusion

In summary, we investigated the levels of chlorophylls, carotenoids, and anthocyanins/flavonoids in four purple-leaf tea cultivars, and explored the potential molecular mechanism underlying purple-leaf formation in tea cultivars through integrated metabolic and gene expression analyses. We found that the purple-leaf phenotype is predominantly attributed to high anthocyanins, as well as low chlorophylls. The purple-leaf phenotype induces changes in other flavonoids, such as reduced levels of monomeric catechin derivatives and increased levels of polymerized catechin derivatives. Gene expression analysis showed that *4CL*, *ANS*, and *UFGT* genes in the anthocyanin biosynthetic pathway and the *HEME* gene in the chlorophyll biosynthetic pathway are responsible for high anthocyanin level and low chlorophyll level, respectively. In addition, the *PSY* gene may be the key gene for carotenoid biosynthesis in the tea leaves. This study facilitates future research on the regulatory mechanism of purple-leaf formation in tea cultivars.

## Data Availability Statement

The original contributions presented in the study are included in the article/Supplementary Material, further inquiries can be directed to the corresponding authors.

## Author Contributions

M-zZ, K-bW, J-aH, and Z-hL designed the study. FZ, L-sR, Y-lL, and BT performed the experiments. M-zZ, FZ, K-bW, J-aH, and Z-hL analyzed the data and wrote the manuscript. All authors read and approved the final manuscript.

## Conflict of Interest

The authors declare that the research was conducted in the absence of any commercial or financial relationships that could be construed as a potential conflict of interest.
