# Supplementary material for: Metabolic Profiling and Gene Expression Analyses of Purple-Leaf Formation in Tea Cultivars (Camellia sinensis var. sinensis and var. assamica)
Source: Front Plant Sci. 2021 Mar 5;12:606962. doi: 10.3389/fpls.2021.606962 (PMC7973281; doi:10.3389/fpls.2021.606962)
Supplement: Supplementary file 1 [file Table_1.docx]

Supplementary Table 1. Primer sequences for qRT-PCR analysis.

| Genes | Forward Primer (5’ - 3’) | Reverse Primer (5’- 3’) |
| --- | --- | --- |
| Glyceraldehyde-3-phosphate dehydrogenase (GAPDH) | TTGGCATCGTTGAGGGTCT | CAGTGGGAACACGGAAAGC |
| Glutamyl-tRNA reductase (HEMA) | CATTTCAACAGGTGTAGTGTGG | CGCTCGAACAACTCAAGGCATC |
| Glutamate 1-semialdehyde aminotransferase (HEML) | GGTTATATAAGCGGGATGTTTG | CAGTGTCACTCTTCTTCGCATC |
| Uroporphyrinogen decarboxylase (HEME) | GAGGAGTCAGTTCCGTATGTTG | AACAAACCCAGTACGGCCGCTT |
| Coproporphyrinogen oxidative decarboxylase (HEMF) | TTGGACTCAAGACGGGAGGTAG | GTTTCCATTCTTCGCTTCCCTC |
| Ferrochelatase (FECH) | AGTTCTTCAACGCTTCCAAGAG | CCACTTTCATTTCAGATTTGGC |
| Mg chelatase H subunit (CHLH) | GGGATGTTTGGGTGAATAGTCC | TTCCTTCCCAAGAAAACCAACC |
| Protochlorophyllide oxidoreductase (POR) | TGACTTGCTCTCTTTGGGGATG | ATCCATGGCTCTCCAGTCTGCT |
| Chlorophyllide a oxygenase (CAO) | ACGTGTCTGGTCCTGTTCAACC | GTGTCATCCTTCAAGTCCGTAG |
| NYC1-like (NOL) | TTTCACGGATGCCATGGAGATG | ATTATGAAGGCACAAACGACAG |
| Phytoene synthase (PSY) | GTTCTTCCGGGGACTTCGAG | GACGCATTAGGCCCATCAAC |
| Phytoene desaturase (PDS) | AGGTGGACAGGCCTATGTTG | TGGCTGGCAAAGTCTCTCTG |
| *ζ*-carotene desaturase (ZDS) | CAGATACAACGGCTGGGTCA | CAGGTGTAAGCACGCATTGG |
| Zeaxanthin epoxidase (ZEP) | TGCAGGAAAGATGCAGTGGT | GTTAAAAGTTGGTGCGCGGT |
| Phenylalanine ammonia lyase (PAL) | CACTCTGCCAAGCCGTAGAT | TGCACTGCAAGGGTCATCAA |
| Cinnamate-4-hydroxylase (C4H) | GAGCATGGACAACAATAGTCTG | ACGTTGTCTTCATTGATCTCTC |
| 4-coumarate: CoA ligase (4CL) | TGACAGAATCAACTGGCGGAATATCG | TGGCTTGGCAATATGGCATCAGAC |
| Chalcone synthase (CHS) | GTGCCATCACATTACAACTTCG | TGAAATGCCTCCACCAAGAT |
| Chalcone isomerase (CHI) | CAGAATTGTTGAAGGAGAAACC | TCAGCAACCTAACCACTAGTGT |
| Flavonoid 3′-hydroxylase (F3′H) | GCAATGATTTCGAGCTCATACC | CTAGTGTGCCCAAAATGTACTC |
| Flavone synthase (FNS) | ACTTCTTGCCTGAGCGATTCTTA | GAAACACTGAATCATTGCCGCT |
| Flavanone-3-hydroxylase (F3H) | CTGGAAACGATGAATCCACCTT | GACTCGCTCGTGAGTTTTTTGC |
| Flavonol synthase (FLS) | CGACGAAGAAGCCAAGACCAAGG | CGGCTCATCAGATCAAGAGGAACAC |
| Dihydroflavonol 4-reductase (DFR) | TCCCATGATGCTACCATCCATG | TCAGTGGGGACATTGTACTCAG |
| Anthocyanidin synthase (ANS) | ACGAGGGCAAATGGGTCA | TCCTTGGGTGGTTCGCAGA |
| Anthocyanidin reductase (ANR) | CCATACCCTCCAGTGCTACGAG | TTGTGAAGATGTTGCTAGAGAG |
| Leucoanthocyanidin reductase (LAR) | CAGAATCATGTTGTTGAAGTAC | AGATGGATTAGATATGGTTCAC |
| UDP-glucose: flavonoid glucosyltransferase (UFGT) | ATTTGGGAACTTAGAATCAGCC | CGTGGTGGTGATGTGAGGTT |
